# Silver nanowires with optimized silica coating as versatile plasmonic resonators

**DOI:** 10.1038/s41598-019-40380-5

**Published:** 2019-03-07

**Authors:** Martin Rothe, Yuhang Zhao, Günter Kewes, Zdravko Kochovski, Wilfried Sigle, Peter A. van Aken, Christoph Koch, Matthias Ballauff, Yan Lu, Oliver Benson

**Affiliations:** 1 Humboldt Universität zu Berlin & IRIS Adlershof, Nanooptics, Newtonstraße 15, 12489 Berlin, Germany; 2 Helmholtz Zentrum Berlin für Materialien und Energie, Institute of Soft Matter and Functional Materials, Hahn-Meitner-Platz 1, 14109 Berlin, Germany; 3 Stuttgart Center for Electron Microscopy, Max Planck Institute for Solid State Research, Heisenbergstr. 1, 70569 Stuttgart, Germany; 4 Humboldt Universität zu Berlin & IRIS Adlershof, Structure Research and Electron Microscopy, Newtonstraße 15, 12489 Berlin, Germany; 5 Humboldt Universität zu Berlin, Department of Physics, 12489 Berlin, Germany; 6 Institute of Chemistry, University of Potsdam, 14467 Potsdam, Germany

## Abstract

Metal nanoparticles are the most frequently used nanostructures in plasmonics. However, besides nanoparticles, metal nanowires feature several advantages for applications. Their elongation offers a larger interaction volume, their resonances can reach higher quality factors, and their mode structure provides better coupling into integrated hybrid dielectric-plasmonic circuits. It is crucial though, to control the distance of the wire to a supporting substrate, to another metal layer or to active materials with sub-nanometer precision. A dielectric coating can be utilized for distance control, but it must not degrade the plasmonic properties. In this paper, we introduce a controlled synthesis and coating approach for silver nanowires to fulfill these demands. We synthesize and characterize silver nanowires of around 70 nm in diameter. These nanowires are coated with nm-sized silica shells using a modified Stöber method to achieve a homogeneous and smooth surface quality. We use transmission electron microscopy, dark-field microscopy and electron-energy loss spectroscopy to study morphology and plasmonic resonances of individual nanowires and quantify the influence of the silica coating. Thorough numerical simulations support the experimental findings showing that the coating does not deteriorate the plasmonic properties and thus introduce silver nanowires as usable building blocks for integrated hybrid plasmonic systems.

## Introduction

In plasmonics, field enhancement effects near metallic nanostructures are utilized to strengthen the light-matter interaction and to miniaturize optical or optoelectronic functionality. Most prominently, plasmonic resonances of metal nanoparticles (NPs) have been used for a plethora of applications and are well understood both experimentally and theoretically^1–11^. However NPs feature some fundamental limitations, e.g., concerning their minimal damping rates or quality (*Q*) factors^10^. Further, frequently studied plasmonic systems are composites of emitters and nanostructures, e.g., for strong coupling^8,12^ or lasing^13–15^. In both scenarios, the envisioned functionalities can be limited by the small physical size of the nanoresonator’s near-field zone, i.e., preventing to host a sufficient number of emitters. Nanowires hold more potential for designing customized nanostructures with both, relatively high Q-factors and yet still small mode volumes, respectively^16,17^. Thus, waveguiding nanostructures, like nanowires (NWs) might be preferred over NPs in many situations. Adjusting cross section and length of NWs allows for intuitive tuning and optimization towards specific needs such as higher Q-factors that go beyond the limits of NPs^18^. Furthermore, plasmonic waveguide structures might be much better suited for integration into dielectric on-chip waveguide structures^19–21^.

Silver nanowires of finite length are especially interesting since they represent ideal type Fabry-Pérot resonators^22,23^. Also silver is well-known for its superior plasmonic properties in the visible spectral range^24^. With respect to the aforementioned composite systems of emitters and nanostructures, it is crucial to control distances on a nm-scale. Besides potential enhancement, metallic nanostructures could also negatively affect nearby emitters due to additional non-radiative channels. The same nm-control is needed to define the distance to nearby materials like additional metallic nanostructures or flat surfaces, e.g., in a wire-on-metal geometry. The surrounding materials shape the mode pattern of plasmonic resonances or guided modes^17^, affecting the field localization as well as damping rates. Also for coupling to integrated dielectric structures, e.g., via directional coupling^25^ or special plasmon-photon transducers^26^ a high accuracy is mandatory. A promising method to reach this goal of precise distance control is a well-defined coating around the metallic nanostructures. Furthermore, for the realization of emitter–resonator composites, a porous dielectric coating like amorphous silica can act as a host for the emitters.

Amorphous silica has been proven an excellent candidate for coating nanostructures, as it is optically transparent, chemically inert and photo-chemically stable^27^. It is also perfectly suited for both emitter-hosting and distance control. While coating with silica shells has been frequently applied to NPs made from gold^27–30^ during the past decade, less work has focused on coating of silver nanostructures like nanowires with amorphous silica shells^31,32^. Silver, however, would provide superior plasmonic properties at shorter wavelengths typical for active nanostructures or in single emitter (quantum) plasmonics^33^. Further, in the reported approaches, the silver is likely to be affected by the used ammonia solution.

In the following, we introduce a novel approach based on a modified Stöber method that improves on these shortcomings for higher quality of the coated silver wires as well of the silica shells. We report on the chemical synthesis and characterization of silver nanowires with a controlled silica-coating, which is well defined in terms of thickness, homogeneity and especially in surface roughness as compared to reports in literature^32,34,35^. The geometry of the silver nanowires is characterized by transmission electron microscopy while UV-VIS, dark-field scattering spectroscopy (DF) and electron energy-loss spectroscopy (EELS) are used to study the influence of the silica coating on the surface plasmon resonances. We measure the effective refractive index of the guided plasmon mode (or the momentum of the surface plasmon polariton (SPP)) and compare it to bare silver nanowires and nanowires with an 8 nm thick silica coating. Further, we employ a Fourier-transformation-based algorithm that allows gaining information about plasmon propagation and damping^36–38^. Finally, we support our findings with numerical Maxwell simulations observing perfect agreement.

## Results and Discussion

### Synthesis and structural analysis

**Figure 1:**
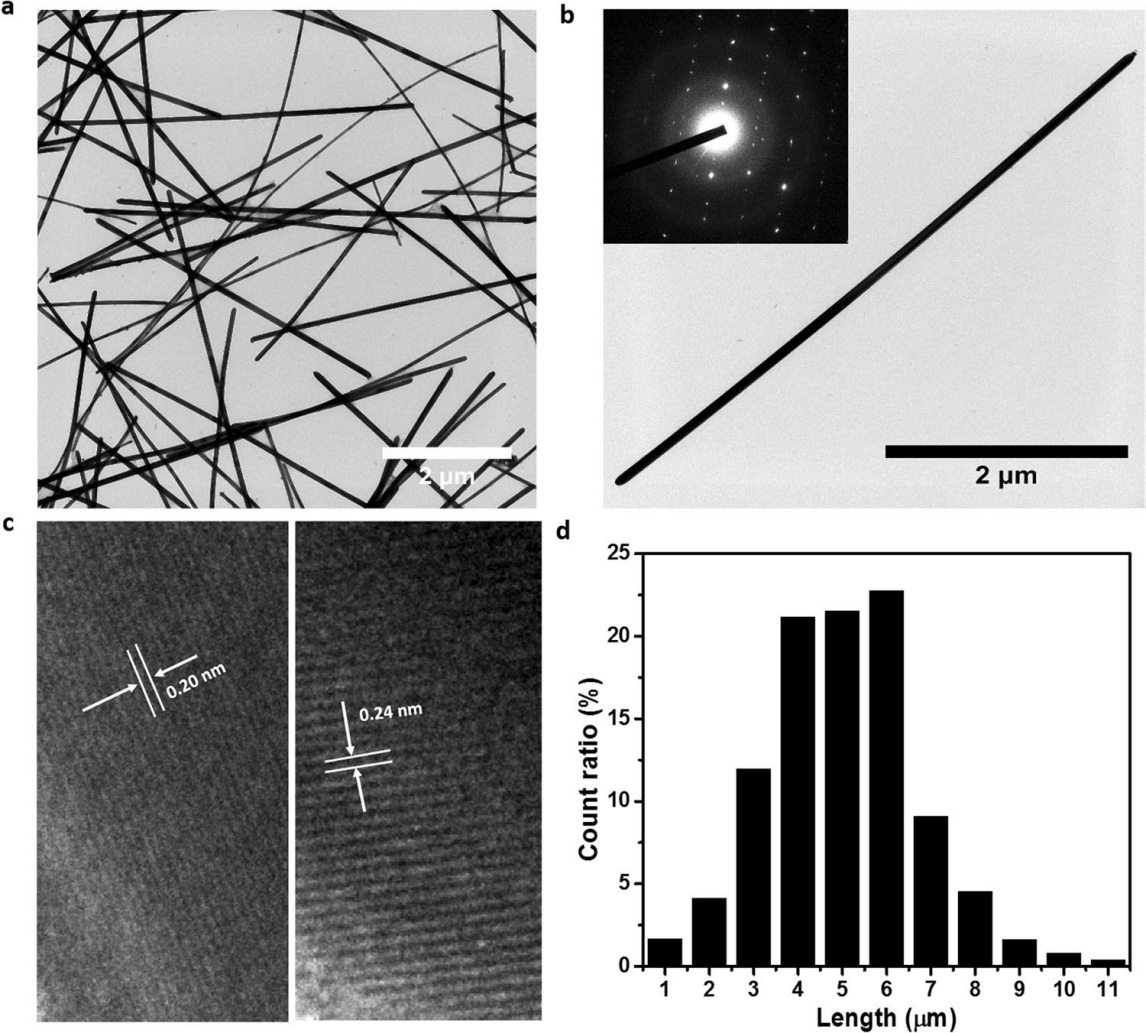
(**a**) TEM image of pure silver nanowires. (**b**) TEM image of a single pure silver nanowire, the inset is the corresponding selected-area electron diffraction pattern. (**c**) High-resolution TEM images taken from each edge of this silver nanowire, indicating the single crystallinity of each side. Lattice spacings of 0.2 nm and 0.24 nm correspond to (200) and (111) planes, respectively. (**d**) Length-distribution histogram of pure silver nanowires counted from TEM micrographs for a particular area.

Silver nanowires were synthesized via a two-injection step in a polyol process^39–41^. Figure 1a shows a typical bright-field TEM image of the as-prepared bare silver nanowires. As can be seen from the image, homogeneous silver nanowires with average length of 5 µm and diameter of 70 nm were fabricated. The distribution histogram of the length of the silver nanowires is shown in Fig. 1d. The inset of Fig. 1b presents an electron diffraction pattern obtained by reducing the size of the electron beam to illuminate only one nanowire, indicating that a twinned crystal structure forms the silver nanowire. The high-resolution TEM images in Fig. 1c reveal that each portion of this twinned nanowire is single-crystalline, and features well-resolved interference fringe spacing^42,43^.

**Figure 2:**
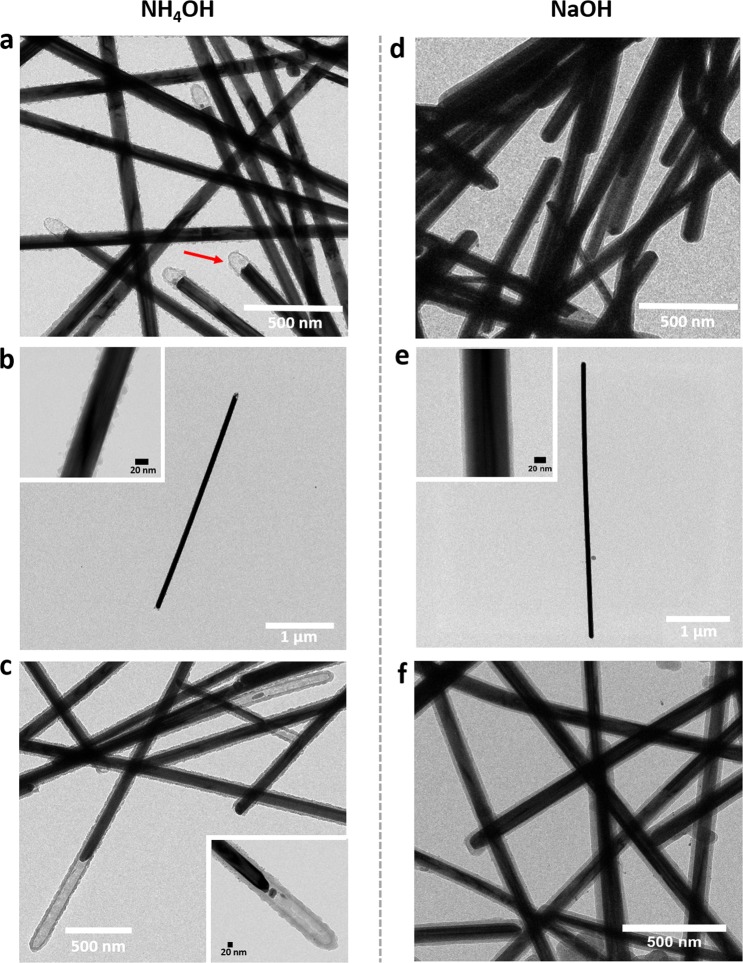
Left column: TEM images of silver nanowires coated with an 8 nm thick silica shell synthesized in ammonia solution: A-SiO_2_8nm@AgNWs (**a**) overview image, (**b**) a single A-SiO_2_@AgNW, the inset shows a magnified insight into the inhomogeneous silica coating structure. (**c**) TEM overview image of A-SiO_2_24nm@AgNWs synthesized in ammonia solution, the inset shows a zoom-in to the etched end of the silver nanowires in silica coating. Right column: TEM images of silver nanowires coated with an 8 nm silica shell synthesized in sodium hydroxide: SiO_2_8nm@AgNWs (**d**) overview image, (**e**) a single composite of SiO_2_8nm@AgNW, the inset shows a magnified insight into the smooth silica coating structure. (**f**) TEM overview images of SiO_2_24nm@AgNWs synthesized in sodium hydroxide solution.

In a next step, we address the coating procedure. So far, coating of the silver nanowires with an amorphous silica shell have been prepared via the Stöber method^31^. In a typical procedure, the encapsulation by a silica shell involves the hydrolysis of tetraethyl orthosilicate (TEOS) and subsequent formation of silica surrounding the silver nanowires. Normally, the hydrolysis of TEOS is performed under an alkaline environment. In the literature, ammonia solution is widely being used to serve as the alkaline condition^44,45^. In our study, we have first coated silver nanowires with an approximately 8 nm thick silica shell assisted by an ammonia solution, as shown in Fig. 2a,b. By increasing the concentration of TEOS from 0.26 vol% to 0.52 vol%, the silica shell thickness increases from around 8 nm to 24 nm. However, although a silica coating was formed by the assistance of the ammonia solution, one can clearly see from the TEM images that the ends of the silver nanowires have been etched by the ammonia solution. Further, also the surface of the silica shells appears wrinkled and uneven. We conclude that ammonia attacks the silver nanowires through the following reaction^45^:

$${\rm{4Ag}}+{{\rm{O}}}_{2}+{\rm{8}}\,{{\rm{NH}}}_{3}\cdot {{\rm{H}}}_{2}{\rm{O}}\to {\mathrm{4Ag}(\mathrm{NH}}_{3}{{)}_{2}}^{+}+{{\rm{4OH}}}^{-}+{{\rm{6H}}}_{2}{\rm{O}}$$

These structural defects might prejudice the optical potential of the well-designed core-shell silver nanowires-based nanostructure. To avoid this phenomenon, we replaced the ammonia solution by sodium hydroxide to provide an alkaline environment for the formation of homogeneous silica shell structures. As can be seen in Fig. 2d,e, silver nanowires with around 8 nm-thick silica shells could successfully be prepared.

As in the case of bare nanowires, single, coated wires are easily identified. Via the improved method, the as-prepared silica surface is very smooth and the silver core of the nanowires maintains its original structure. By increasing the TEOS concentration from 0.26 vol% to 0.52 vol%, silica shells with a thickness of 24 nm were achieved uniformly covering the entire surface of the silver nanowire without etching the silver nanowire’s core structure (see Fig. 2f). This indicates that an excellent control of the thickness of the silica shell can be achieved by changing the concentration of TEOS. These results highlight the potential of the improved silica coating method to achieve silver nanowires with homogeneous and thickness-controllable silica shells.

**Figure 3:**
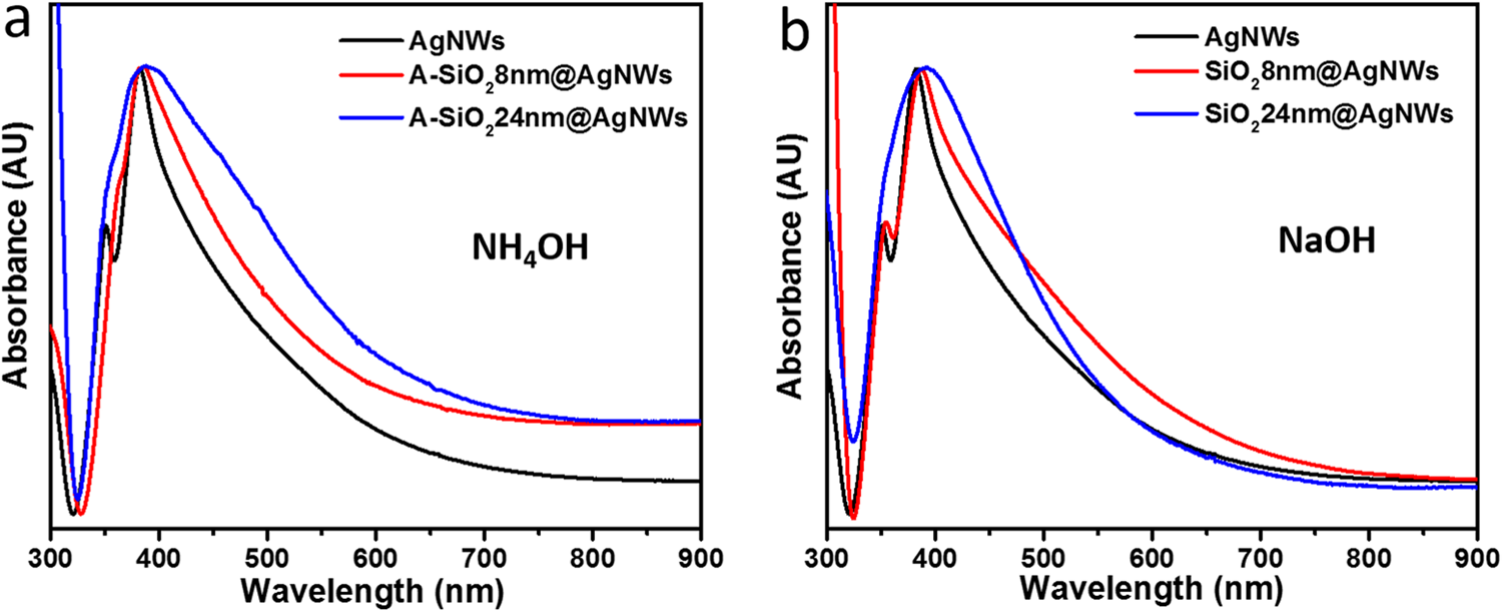
UV-VIS absorption spectra of pure silver nanowires and silica coated silver nanowires synthesized in different alkaline environment: (**a**) ammonia solution, (**b**) sodium hydroxide. The peaks at 350 nm and 382 nm are ascribed to the bulk and the transversal plasmon, respectively.

We now focus on studying the optical properties by means of UV-VIS ensemble measurements of the as-grown nanowires and of the coated nanowires using ammonium and sodium hydroxide. Figure 3 shows the UV-VIS spectra of silver nanowires dispersed in aqueous solution with a standard ultraviolet-visible absorption of pure silver nanowires showing the dominant characteristic features of silver nanowires, i.e. one smaller peak at around 350 nm and a maximum at around 382 nm. These peaks stem from localized surface plasmon resonances, as reported in literatures^23,46,47^, and are effected by the pentagonal cross-section of the nanowires, together with relatively strong corner-rounding. The red-shifting of the spectrum with increasing silica-shell thickness arises from the local change of the refractive index^46^. An additional broad shoulder appears in the tail between 400 nm and 650 nm, which results from the distribution of possible orientations of the nanowires with respect to the incident light. Additional minor lifting of the red range of the spectrum is attributed to coupling to travelling plasmons along the nanowire and coagulation of particles, also known from spherical nanoparticles^48^. For the coated AgNWs we find that the dominant spectral features are broadened for the thickest coating of around 24 nm. For the thinner coating of around 8 nm we find that the ammonium treated AgNWs show a modified first surface plasmon peak, while the sodium hydroxide treatment leaves this peak rather unaffected. Thus, we take these ensemble measurements as further indication that the sodium hydroxide indeed represents a gentler treatment than the treatment with ammonium.

### Study of individual AgNWs

**Figure 4:**
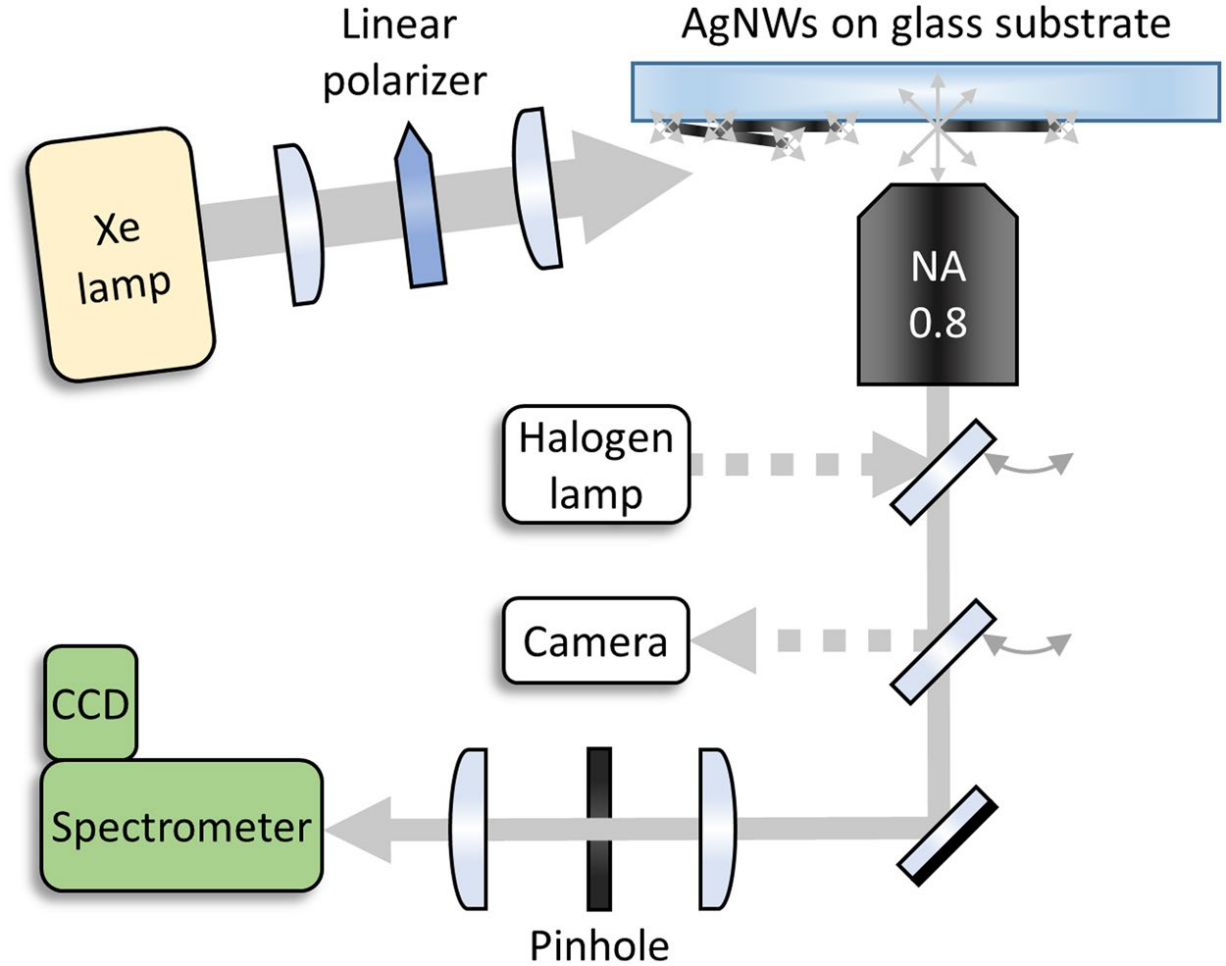
Experimental setup: Optical measurements on single nanowires with a home-build microscope. A halogen lamp is used for bright-field illumination. A Xenon arc lamp provides white light, which is subsequently linearly polarized and sent from the side to create a dark-field configuration. Scattered light is filtered spatially by focusing on a pinhole and detection by a spectrometer.

To study the quality of the sodium hydroxide treated AgNWs in more detail we now turn to our studies on individual AgNWs. A clear indication of a good quality in plasmon resonators is the appearance of standing wave resonances. For this reason, we look for and analyze such Fabry-Pérot-type resonances in our silver wires. In order to observe the influence of the silica coating on the plasmonic mode of individual silver nanowires, silver nanowires with 8 nm and 24 nm silica coating were prepared on cover glass by spin coating, respectively. The resulting randomly distributed nanowires could then be studied one-by-one in our home-build microscope setup (Fig. 4).

**Figure 5:**
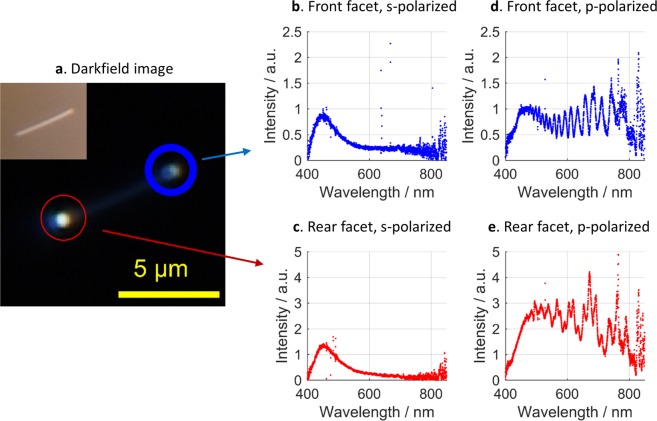
A single silver nanowire detected by dark-field (**a**) and bright-field (inset) imaging. Both, the front facet (**b**) and the rear facet (**c**) show a broad resonance in the scattering spectrum under s-polarized illumination. Under p-polarized illumination, longitudinal plasmon modes are excited. Thus, the scattered light reveals Fabry-Pérot resonances most clearly visible for scattering at the front facet (**d**). The rear facet (**e**) shows similar features, but overlaid with interference with the incident light.

Bright-field imaging (Fig. 5a inset) allows for detection of single nanowires. Subsequently we use dark-field illumination with linear polarized light from a Xenon arc lamp to analyze the scattering properties of the nanowires. We focus on those nanowires, which lie aligned parallel to the direction of propagation of the incident light, as these show strong scattering only from their front and rear facet and thus less background (Fig. 5a). We spatially separate light from each end facet by the confocal principle and guide it to the spectrometer. The obtained scattering spectra were background subtracted and normalized to the measured and background-subtracted spectrum of the white light source.

Typical normalized scattering spectra from a single silver nanowire are shown in Fig. 5b–e. Selecting the polarization of the incident light allows addressing distinct plasmonic modes. Furthermore, one has to distinguish between the front and the rear facet scattering, as additional interference effects might be observed that are not necessarily stemming from the Fabry-Pérot interferometer formed by the individual AgNWs. For p-polarized illumination, the spectrum of the front facet shows strong oscillations (Fig. 5d), that is a clear signature of coupling between the white light and the longitudinal plasmon modes. We find that the spectra can be modeled as the reflection of a Fabry-Pérot resonator^38,49^. According to the relatively strong damping in such a plasmonic resonator the expected spectral features like peaks and dips broaden and follow a sinusoidal behavior. When the polarization is changed to s-polarization (Fig. 5b,c) the oscillation vanishes towards a broad peak in the blue range of the spectrum. In this case, no coupling to longitudinal modes is expected due to the symmetry of these modes. The broad peak stems from scattering by the short axis of the wire, i.e. it is a signature of the transversal mode. This feature is also present in the spectra recorded with p-polarization as the used polarization filter has limited performance for the blue side of the spectrum.

At the rear facet we find again an oscillating spectrum when using p-polarized illumination (Fig. 5e), however with an additional modulation. This is due to a superposition of two contributions caused by the simultaneous illumination of both facets. One contribution is the direct scattering from the rear facet, the other one stems from light that was transmitted through the wire and then eventually scattered into photonic modes. Both, reflection and transmission spectra of a Fabry-Pérot resonator carry the same information about the resonator. Since the above mentioned superposition does not occur in the front facet spectra, these spectra are used for further analysis of the resonator. Our analysis yields the real part of the effective refractive index Re(*n*_eff_) of the guided mode and the plasmon round trip loss. The latter contains propagation losses α and losses due to imperfect end facet reflectivities *R*.

**Figure 6:**
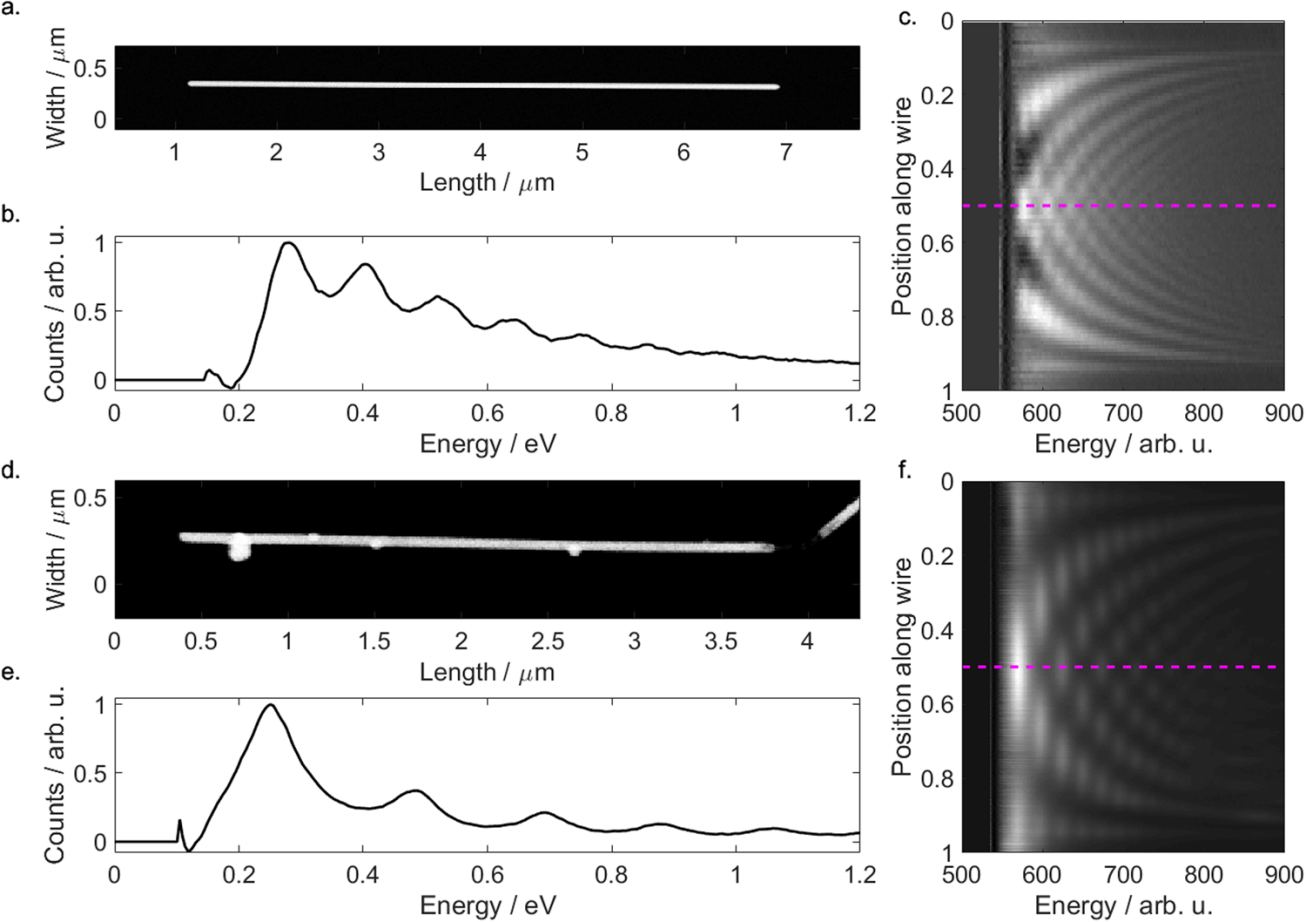
Summary of EELS measurements of an as-grown nanowire (**a**–**c**) and a coated nanowire with 8 nm silica shell (**d**–**f**). (**a,d**) TEM images of nanowires, (**b,e**) EELS spectrum recorded next to the nanowire’s center as indicated by the dashed line in (**c**) and (**f**), respectively. (**c,f**) Spectral EELS maps recorded along the nanowires: horizontal lines correspond to spectra at fixed positions, while vertical lines correspond to standing wave pattern for fixed loss energies.

While dark-field spectroscopy in principle works nicely for nanoparticles, it may not unambiguously exclude any source of interference that might only mimic Fabry-Pérot resonances for larger structures^50,51^. Thus, we further performed complementary EELS measurements of AgNW specimen, as EELS directly provides maps of the projected local optical density of states of standing wave patterns of the resonating field along the AgNW resonator. Figure 6 summarizes our findings where we recorded a set of EELS spectra for varying positions along coated and as-grown nanowires.

### Quantitative analysis of plasmonic losses

**Figure 7:**
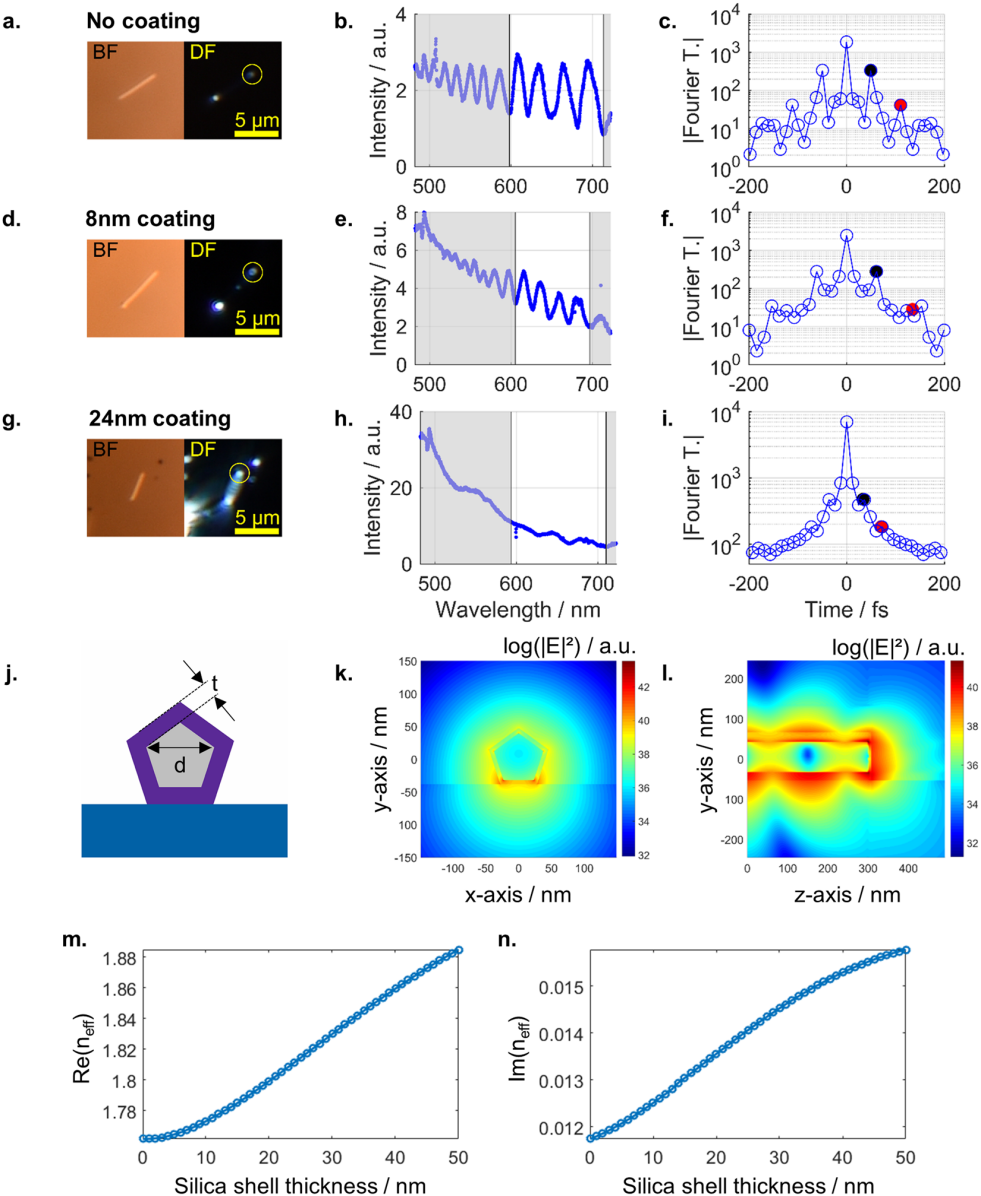
Detailed quantitative analysis of the influence the silica shell. Bright-field (BF) and dark-field (DF) images of single nanowires, measured scattering spectra of light from the front facets and Fourier transformation of the spectra (in the frequency domain) around 650 nm (white area in middle column) of nanowires without (**a**–**c**), with 8 nm (**d**–**f**) and 24 nm thick silica shell (**g**–**i**). The plasmon round trip time (black dot) and the second round trip time at twice the time (red dot) are indicated. The cross section (**j**) of the silver nanowire of diameter *d* coated with a silica shell of thickness *t* is used to simulate the guided plasmon mode (**k**) for λ_0_ = 650 nm. A full 3D computation yields the logarithmic intensity distribution (cut through the center of the wire) at the end facet (**l**) and the reflectivity. The computation further yield the real part (**m**) and the imaginary part (**n**) of the effective refractive index of the guided mode depending on the shell thickness.

We now move on with a quantitative analysis of our data. The most important parameter to judge the quality of our plasmonic nanostructures is the loss rate or round trip loss (RTL). We experimentally derive this number by analyzing the spectra utilizing a fast Fourier transformation (FFT). Figure 7 presents bright-field and dark-field images together with scattering spectra and FFT spectra, for example of an as-grown AgNW (Fig. 7a), a AgNW with 8 nm silica coating (Fig. 7d) and a AgNW with 24 nm silica coating (Fig. 7g). We point out here that the preparation of the nanowires with 24 nm shells was difficult due to strong clustering of this type of nanowire in dispersion before the spin coating preparation. Stronger and longer sonification dissolved the clusters, but also led to shorter AgNWs due to NW fracture. Plasmonic resonances of shorter NWs are harder to study sufficiently. On the one hand, scattering from the two adjacent ends can interfere in the detectors. On the other hand, the free spectral range of shorter NWs is larger, making it necessary to evaluate an inaccessible larger spectral range or losing information.

We selected a wavelength range of roughly 100 nm around the center wavelength of 650 nm (white area in the second column of Fig. 7) for further analysis, i.e., the spectral range for which we found the best signal to noise ratio. The selected range was converted to the frequency space and a FFT was performed to analyze the resonator properties round trip time and damping^36–38^. The boundaries of the spectral window were selected to coincide with a local minimum or maximum to improve the quality of the FFT. Furthermore, only spectra with at least two oscillations where considered.

The Fourier transforms are presented in the right column of Fig. 7 in the range of −200 to 200 fs. The full range Fourier transformations are available in the supplementary. Note that the resolution of the Fourier transformation is limited by the number of data points and the range of the analyzed spectrum. As already shown by Alione *et al*.^38^ and Hofstetter *et al*.^36,37^, the position of the first peak (black dot) in the sidebands of the Fourier transformation contains directly the information of the plasmon round trip time. For each nanowire the peak position was extracted by fitting the data in the vicinity of the discrete maximum with a parabolic function. The apex position was assigned to be the plasmon round trip time *T*_rt_ in the nanowire. The length *L* of the nanowire is measured from the dark-field images. Compared to the speed of light in vacuum, c, the plasmonic wave travels more slowly by a factor of Re(*n*_eff_)^52^. Within T_rt_ the wave travels twice the length of the nanowire, 2 L, leading to

1 $${\rm{Re}}({n}_{{\rm{eff}}})=\frac{c\cdot {T}_{{\rm{rt}}}}{2L}$$

In order to compare the experimental findings with theory, we performed numerical simulations with a finite element solver (JCMsuite, see method section for details) for a fixed frequency corresponding to a vacuum wavelength of *λ*_0_ = 650 nm. This wavelength was chosen, as it is centered in the experimentally evaluated range. The real part of the calculated effective refractive index *n*_eff_ of the fundamental propagating mode (Fig. 7k) contains information about the wavelength of the propagating plasmon polariton $({\lambda }_{{\rm{s}}{\rm{p}}{\rm{p}}}={\lambda }_{0}/{\rm{R}}{\rm{e}}({n}_{{\rm{e}}{\rm{f}}{\rm{f}}}))$, whereas the imaginary part is related to its damping constant $(\alpha =4\pi \cdot {\rm{Im}}({n}_{{\rm{eff}}})/{\lambda }_{0})$. Subsequently, the propagating mode was launched into a three-dimensional (3D) computational domain to evaluate the reflectivity R of the end facets of a semi-infinite silver nanowire (Fig. 7l). In this 3D simulation, we restrict ourselves to flat end facets for simplicity. In simulations without any substrate (isotropic surrounding around the wire and cylindrical symmetry), we found that the shape of the end facet (tapered tip) has almost no impact on its reflectivity. The results for the real and the imaginary part of *n*_eff_ are plotted in Fig. 7m,n, respectively.

**Figure 8:**
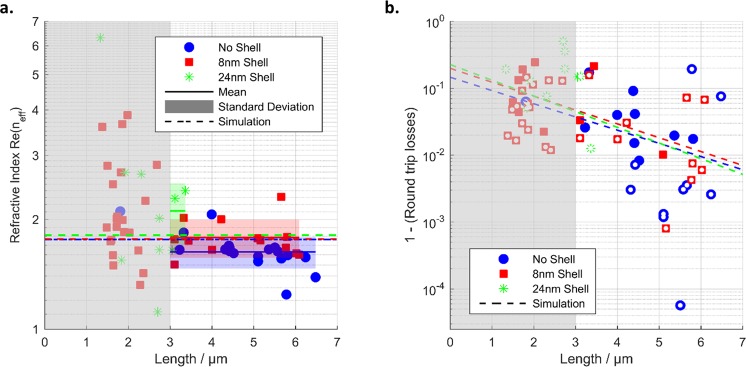
(**a**) Real parts of the effective refractive index Re(*n*_eff_) as experimentally derived (scattered markers) and theoretically predicted (dashed lines). Solid lines and shaded areas (in green, red and blue) are mean and standard deviation of each group of nanowires (without shell, with 8 nm shell, with 24 nm shell), respectively. Note that our method becomes reliable (see text) for nanowires longer than 3 µm (white area). (**b**) Survival probability *P*_survival_ as extracted from Fourier spectra for the nanowire specimens (markers). Full markers represent data where a clear second round trip peak could be observed, while hollow markers represent data where only an upper limit could be deduced. The dashed lines show the results of the numerical calculation.

Figure 8 summarizes our experimental findings with a comparison to theoretical predictions. Figure 8a shows a scatter plot of the measured Re(*n*_eff_) as a function of the corresponding nanowire length *L* of the investigated specimen together with the values found in our numerical simulations (indicated by straight horizontal lines as *n*_eff_ is independent of the waveguide length). We find that relatively short nanowires show a larger variance of Re(*n*_eff_) which we attribute to general limitations of the applied dark-field microscopy. Nanowires with short lengths close to the diffraction limit suffer from three uncertainties: First, the relative error of the measured nanowire lengths is large for short nanowires. Second, the front facet and end facet are spatially not well separated so that contributions from both ends might enter the detection. Further, the free spectral range of short nanowires is relatively large so that only a few oscillations are apparent in the scattering spectrum. With respect to our analysis based on a Fourier transformation this means that the central peak will be very close to the peak representing one roundtrip time, which is thus hard to resolve.

However, we find that for nanowires longer than 3 µm (white area in Fig. 8a), the method becomes reliable. The variance reduces significantly and the experimentally derived Re(*n*_eff_) scatter around the theoretically expected value (dashed line Fig. 8a). We further included the mean and standard deviation of Re(*n*_eff_) for the nanowires longer than 3 µm as solid lines and as colored shaded areas for the coated and uncoated nanowires, respectively. The simulated values are covered by the standard deviations. From this we conclude that the silica shell has only minor influence on Re(*n*_eff_) as already predicted by our simulations.

More importantly, the plasmon damping can be extracted from the data. An additional peak is expected in the Fourier spectrum at twice the round trip time (*t* = 2*T*_rt_). The harmonic peak amplitude ratio, i.e., the ratio of the second peak *p*_2_ and the first peak *p*_1_, is a direct measure of the overall round trip losses *RTL*^36–38^. These losses stem from imperfect reflectivities *R*_1_ and *R*_2_ of the end facets and the damping constant α of the SPPs. Besides sole Ohmic damping of the SPP, the apparent α may contain losses by scattering, e.g., due to roughness of the metal-dielectric interface of the AgNWs or imperfections of the silica coating. Reflectivities and damping are connected to the harmonic amplitude ratio by the survival probability *P*_survive_

2 $${{P}}_{{\rm{survive}}}=1-{\rm{RTL}}={(\frac{{{p}}_{2}}{{{p}}_{1}})}^{2}={({R}\cdot {{e}}^{-{\boldsymbol{\alpha }}L})}^{2}$$

i.e., the ratio of SPPs surviving one round trip^36–38^. Here we assume equal reflectivities at both facets $(R=\sqrt{{R}_{1}{R}_{2}})$. The relatively high round trip losses correspond to small peak heights *p*_2_ in the Fourier spectra as can be seen in Fig. 7 where the black dot marks *p*_1_ and the red dot *p*_2_ (on a log scale). The offset, overlying tilt, and noise in the scattering spectrum leads to a background in the Fourier spectra, while SPP damping and dispersion leads to a broadening of the peaks. Thus, the peak height *p*_2_ could not be extracted for all nanowires, but we only deduced an upper limit for the harmonic amplitude ratio or *P*_survive_, respectively. In Fig. 8b, we show *P*_survive_ versus the corresponding length of the nanowire specimen. Solid points represent data, where a second peak could be clearly identified, while hollow points indicate situations, where only an upper limit could be estimated. For the nanowires without coating, we find the longest specimen. Half of investigated specimens (9 out of 19) of length greater than 3 µm show a pronounced second peak in the Fourier spectra (solid blue points). For the nanowires with silica coating of 8 nm, 3 out of 13 Fourier spectra show a pronounced peak height *p*_2_ (solid red boxes in Fig. 8b). For these specimens *P*_survive_ is comparable to the nanowires without coating. For the remaining ten spectra, we again interpret the results as upper bounds (hollow red boxes in Fig. 8b). Additionally, we were able to extract *P*_survive_ of a single nanowire with 24 nm silica coating. Together with this scattered data, we plot the theoretically expected *P*_survive_ derived from the numerical simulations. These findings suggest that the silica coating is doing no harm to the bare AgNWs and the plasmonic performance. Though, we had to sonicate coated AgNWs for longer times to avoid agglomeration, which led to more broken, and thus on average shorter nanowires. However, we believe that this problem can be avoided, e.g., by more diluted samples that can be spin-casted multiple times to achieve equal densities on substrates. For the sample with the thickest coatings we observed secondary silica particles, partly attached to the AgNWs, which limits the method somehow as these secondary particles act as efficient scatterers of the SPP field representing additional loss channels. Nevertheless, we believe that again the synthesis procedure could be optimized to avoid such secondary particles more efficiently.

**Figure 9:**
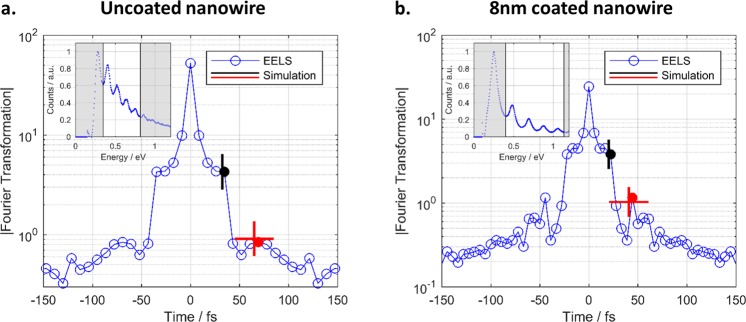
Detailed analysis of the EELS spectra of (**a**) the uncoated and (**b**) the 8 nm coated AgNW. The Fourier transformations reveal the expected peaks at the first round trip time (black dot) and the second round trip time (red dot). Both position and peak amplitude ratio match the simulation (black and red lines). The inset indicates the analyzed part of the EELS spectra (white area).

Finally, our findings are also nicely supported by the evaluation of the EELS spectra. Firstly, the EELS data clearly show the standing wave pattern expected for the plasmonic Fabry-Pérot resonators. This further supports our understanding that we indeed probe the Fabry-Pérot resonances with our optical studies and do not observe oscillating spectra that are just due to accidentally formed micro-resonators^34^. Here, the EELS spectra could be evaluated best in the infrared spectral range, i.e., for relatively small electron energy loss. In Fig. 6, several harmonics are clearly visible for different electron loss energies for both a coated and an uncoated nanowire. Applying the same Fourier algorithm to the center-line spectrum (Fig. 6b,e, horizontal line) we again create a Fourier spectrum (Fig. 9a,b) for which we apply the same analysis as for the optical investigations. The real part of the effective refractive index were found to be 1.70 and 1.98, for the uncoated and coated AgNWs, respectively. This is in agreement to simulated values of 1.59 and 1.82. The position of the second peak is indicated by the red dot. *P*_survival_ is estimated to be 0.039 and 0.092 from this. These values also match the simulated results of 0.043 and 0.095, calculated from the imaginary part of the refractive index of 0.011 and 0.017 and reflectivities of 0.34 and 0.46, respectively. The simulated first and second round trip time is shown in Fig. 9 as black and red vertical line, respectively. The simulated damping results in an expectation of the second peak height (horizontal red line). Both matching peak position and height illustrates the excellent agreement between our measurements and simulations. This supports the conclusion that especially the damping of the SPPs is barely affected by the silica coating.

## Conclusion

We reported on the synthesis of thin and short silver nanowires (70 nm in diameter and few µm in length) on which we apply a modified Stöber method to create an optimized silica coating (here 8 nm and 24 nm thick). Such coatings enable functionalization with emitters and distance control. We use transmission electron microscopy, single-particle dark-field scattering spectroscopy and thorough numerical calculations to demonstrate that the modified Stöber method yields well-defined smooth silica coatings that do no harm to the plasmonic properties of the silver nanowires.

We conclude that our modified synthesis method opens the door for the construction of well-defined coupled systems of quantum emitters and plasmonic resonators and their usage in integrated optical devices^19–21^, i.e. all-optical transistors^53,54^ or active plasmonic sensors^55^. Such coatings have been frequently used for gold nanoparticles so far but are now accessible to silver nanostructures like nanowires, too. We thus expect our work to have a broad impact on future studies on hybrid plasmonic systems based on silver nanostructures.

With respect to surface plasmon lasing^13–15^ or strong coupling experiments^8,12^, we believe that the geometric advantage of nanowire modes to be able to overlap spatially with much more gain medium (or emitters) compared to nanoparticle modes can now be accessed much more conveniently as the silica coating allows for incorporation of emitters^56^.

## Method

### Materials

Silver nitrate (99%), ethylene glycol (99.8%), poly(vinyl pyrrolidone) (Mw = 40000), tetraethyl orthosilicate (TEOS), sodium hydroxide, sodium chloride, ammonia solution (28 wt%), absolute ethanol were purchased from Aldrich. All the chemicals were used without further purification. Cover glass slips were cleaned with Hellmanex solution.

### Synthesis of silver nanowires

The synthesis of silver nanowires followed the method develop by Robert K. Y. Li^39^. In a typical run, 2.005 g poly(vinyl pyrrolidone) (Mw = 40000) was dissolved in 34 ml ethylene glycol under heating and stirring. After the temperature was stable at 160 °C, 40 µl NaCl solution (0.8 mM) was added into the flask. One minute later, 6 ml AgNO_3_ (0.1 M) in ethylene glycol solution was injected by a syringe pump at a flow rate of 10 µl s^−1^. Once the reaction solution turned turbid, all residual AgNO_3_ solution was added into the flask immediately. The mixture was then continuously stirred for 30 min at 160 °C followed by natural cooling down to ambient temperature. The as-prepared AgNWs were collected via centrifugation and stored in ethanol at 4 °C.

### Silica coating of silver nanowires

For the silica coating, 35 µl AgNWs solution (6 mg ml^−1^) was dispersed in the mixture of 6 ml absolute ethanol, 2 ml deionized water and 200 µl NaOH (0.1 M) under magnetic stirring. To gain silver nanowires with 8 nm silica shell, 1 ml TEOS solution (0.26 vol% of TEOS in ethanol) was added slowly with continuous stirring for 15 min. The mixture was stirred at 500 rpm for further 3 h at ambient temperature. As-prepared silica coated silver nanowires (noted as SiO_2_8nm@AgNWs) were collected via centrifugation after rinsed in water. Silver nanowires with around 24 nm silica shell (noted as SiO_2_24nm@AgNWs) were achieved by tuning the concentration of TEOS to 0.52 vol% keeping the other conditions the same. As a comparison, 200 µl NaOH (0.1 M) were replaced by 200 µl ammonia solution (28 wt%) under the same condition. The pH values of the solution treated with ammonia and NaOH are 11.8 and 12.5, respectively. Samples of silica coated silver nanowires with around 8 nm and 24 nm shell are noted as A-SiO_2_8nm@AgNWs and A-SiO_2_24nm@AgNWs correspondingly.

### Characterization

The morphology of the nanowires was investigated by transmission electron microscopy (TEM) using a JEOL JEM-2100 (JEOL GmbH, Eching, Germany) at an acceleration voltage of 200 kV. Samples were prepared by placing a drop of the diluted nanowires solution on the carbon-coated copper grids and dried under ambient temperature. The ultraviolet-visible spectra (UV-VIS spectra) were measured by Lambda 650 spectrometer supplied by Perkin-Elmer or Agilent 8453.

### Bright-field and dark-field optical microscopy

The plasmonic modes of individual nanowires were analyzed by white-light scattering in a home-build microscope setup. Samples were prepared on cleaned cover-glass slips from nanowire dispersions which were diluted with ethanol and placed into an ultrasonic bath for 5 to 15 minutes. Subsequently, 50 to 150 µl of the dispersion were spin-coated onto a clean cover glass slip. This procedure was optimized for every dispersion to achieve a convenient distribution of single nanowires on the cover glass. Single nanowires were imaged by bright-field and dark-field illumination using a Nikon D5500 camera. A Xenon arc lamp equipped with a linear polarizer was used for illumination.

Scattered light was analyzed in a confocal setup with an Andor Newton DU-920P-BV CCD-camera mounted on an Acton Spectra Pro 2500i spectrometer.

### Numerical simulation

We modeled the plasmonic Fabry-Perot resonator with a finite element Maxwell’s equations solver (JCMsuite) in the frequency domain. All calculations were done for a vacuum wavelength of 650 nm. For silver we used a refractive index n_ag_ = 0.0535 + i⋅4.4107^24^ and assumed the cross-section of the nanowires to be a regular pentagon^57^. The silica shell around the metallic nanowire was assumed to have a refractive index of 1.4. The (coated) nanowire laid directly on a glass substrate of a refractive index set to 1.4565. For all simulations we used perfectly matched layers as boundaries. The field distribution of the fundamental propagating mode was computed together with the corresponding complex effective refractive index *n*_eff_. In order to compute the reflectivity R of the end facet we used the field computed with the propagating mode solver as a source that was launched into a three-dimensional (3D) computational domain with a semi-infinite silver nanowire. The result was obtained by setting all imaginary parts of the contributing permittivities to zero and computing the ratio of the reflected flux with the launched flux.

### Electron energy-loss spectroscopy

Measurements of the plasmonic response were conducted in the Zeiss SESAM microscope operated at a high tension of 200 kV. Using an electrostatic electron monochromator, this instrument offers an energy resolution below 100 meV. EELS spectra were recorded at a dispersion of 0.0047 eV/pixel using the in-column MANDOLINE energy filter. 100 or 400 spectra were collected along the edge of the wire with a dwell time of typically 0.5 s per spectrum. The electron beam was always outside the wire (aloof geometry) to minimize volume contributions to the spectra. High-angle annular dark-field (HAADF) images were acquired for scattering angles ranging from 28 to 190 mrad.

## Supplementary information

Supporting Information Silver nanowires with optimized silica coating as versatile plasmonic resonators

## Data Availability

All data generated or analyzed during this study are included in this published article (and its Supplementary Information files).

## References

[1] Schuller JA (2010). Plasmonics for extreme light concentration and manipulation. Nature Materials.

[2] Barnes WL, Dereux A, Ebbesen TW (2003). Surface plasmon subwavelength optics. Nature.

[3] Kelly KL, Coronado E, Zhao LL, Schatz GC (2003). The Optical Properties of Metal Nanoparticles:  The Influence of Size, Shape, and Dielectric Environment. The Journal of Physical Chemistry B.

[4] Brongersma ML, Halas NJ, Nordlander P (2015). Plasmon-induced hot carrier science and technology. Nature Nanotechnology.

[5] Zhang JZ (2010). Biomedical Applications of Shape-Controlled Plasmonic Nanostructures: A Case Study of Hollow Gold Nanospheres for Photothermal Ablation Therapy of Cancer. The Journal of Physical Chemistry Letters.

[6] Novotny L, van Hulst N (2011). Antennas for light. Nature Photonics.

[7] Baffou G, Quidant R (2013). Thermo-plasmonics: using metallic nanostructures as nano-sources of heat. Laser & Photonics Reviews.

[8] Törmä P, Barnes WL (2015). Strong coupling between surface plasmon polaritons and emitters: a review. Reports on Progress in Physics.

[9] Ziegler J, Djiango M, Vidal C, Hrelescu C, Klar TA (2015). Gold nanostars for random lasing enhancement. Opt Express.

[10] Moroz A (2010). Non-radiative decay of a dipole emitter close to a metallic nanoparticle: Importance of higher-order multipole contributions. Optics Communications.

[11] Ruppin R (1982). Decay of an excited molecule near a small metal sphere. The Journal of Chemical Physics.

[12] Wersall M, Cuadra J, Antosiewicz TJ, Balci S, Shegai T (2017). Observation of Mode Splitting in Photoluminescence of Individual Plasmonic Nanoparticles Strongly Coupled to Molecular Excitons. Nano Lett.

[13] Yang, A., Wang, D., Wang, W. & Odom, T. W. Coherent Light Sources at the Nanoscale. *Annu Rev Phys Chem* (2017). 10.1146/annurev-physchem-052516-050730 28142312

[14] Hill MT, Gather MC (2014). Advances in small lasers. Nature Photonics.

[15] Oulton RF (2009). Plasmon lasers at deep subwavelength scale. Nature.

[16] Kewes, G. *et al*. Limitations of Particle-Based Spasers. *Physical Review Letters* **118** (2017). 10.1103/PhysRevLett.118.237402 28644673

[17] Oulton RF, Sorger VJ, Genov DA, Pile DFP, Zhang X (2008). A hybrid plasmonic waveguide for subwavelength confinement and long-range propagation. Nature Photonics.

[18] Johns P, Beane G, Yu K, Hartland GV (2017). Dynamics of Surface Plasmon Polaritons in Metal Nanowires. The Journal of Physical Chemistry C.

[19] Gramotnev DK, Bozhevolnyi SI (2010). Plasmonics beyond the diffraction limit. Nature Photonics.

[20] Fang Y, Sun M (2015). Nanoplasmonic waveguides: towards applications in integrated nanophotonic circuits. Light: Science &Amp; Applications.

[21] Xin G, Yaoguang M, Yipei W, Limin T (2013). Nanowire plasmonic waveguides, circuits and devices. Laser & Photonics Reviews.

[22] Schell AW, Kuhlicke A, Kewes G, Benson O (2017). “Flying Plasmons”: Fabry-Pérot Resonances in Levitated Silver Nanowires. ACS Photonics.

[23] Rossouw D, Botton GA (2013). Plasmonic Response of Bent Silver Nanowires for Nanophotonic Subwavelength Waveguiding. Physical Review Letters.

[24] Johnson PB, Christy RW (1972). Optical Constants of the Noble Metals. Physical Review B.

[25] Degiron A, Cho S-Y, Tyler T, Jokerst NM, Smith DR (2009). Directional coupling between dielectric and long-range plasmon waveguides. New Journal of Physics.

[26] Kewes G (2013). Design and numerical optimization of an easy-to-fabricate photon-to-plasmon coupler for quantum plasmonics. Applied Physics Letters.

[27] Rodríguez-Fernández J, Pastoriza-Santos I, Pérez-Juste J, García de Abajo FJ, Liz-Marzán LM (2007). The Effect of Silica Coating on the Optical Response of Sub-micrometer Gold Spheres. The Journal of Physical Chemistry C.

[28] Li JF (2010). Shell-isolated nanoparticle-enhanced Raman spectroscopy. Nature.

[29] Liu S, Zhang Z, Wang Y, Wang F, Han M-Y (2005). Surface-functionalized silica-coated gold nanoparticles and their bioapplications. Talanta.

[30] Kobayashi Y, Correa-Duarte MA, Liz-Marzán LM (2001). Sol−Gel Processing of Silica-Coated Gold Nanoparticles. Langmuir.

[31] Stöber W, Fink A, Bohn E (1968). Controlled growth of monodisperse silica spheres in the micron size range. Journal of Colloid and Interface Science.

[32] Fedutik Y, Temnov V, Woggon U, Ustinovich E, Artemyev M (2007). Exciton−Plasmon Interaction in a Composite Metal−Insulator−Semiconductor Nanowire System. Journal of the American Chemical Society.

[33] Kumar S, Davydov VA, Agafonov VN, Bozhevolnyi SI (2017). Excitation of nanowire surface plasmons by silicon vacancy centers in nanodiamonds. Opt. Mater. Express.

[34] Achtstein AW, Schoeps O, Artemyev MV, Woggon U (2010). Excitonic properties of single CdSe nanowires and coupling to plasmonic nanocavities. physica status solidi (b).

[35] Fedutik Y, Temnov VV, Schops O, Woggon U, Artemyev MV (2007). Exciton-plasmon-photon conversion in plasmonic nanostructures. Phys Rev Lett.

[36] Hofstetter D, Thornton RL (1997). Theory of loss measurements of Fabry–Perot resonators by Fourier analysis of the transmission spectra. Optics Letters.

[37] Hofstetter D, Thornton RL (1998). Measurement of optical cavity properties in semiconductor lasers by Fourier analysis of the emission spectrum. IEEE Journal of Quantum Electronics.

[38] Allione M, Temnov VV, Fedutik Y, Woggon U, Artemyev MV (2008). Surface Plasmon Mediated Interference Phenomena in Low-Q Silver Nanowire Cavities. Nano Letters.

[39] Hu M, Gao J, Dong Y, Yang S, Li RKY (2012). Rapid controllable high-concentration synthesis and mutual attachment of silver nanowires. RSC Advances.

[40] Sun Y, Yin Y, Mayers BT, Herricks T, Xia Y (2002). Uniform Silver Nanowires Synthesis by Reducing AgNO3 with Ethylene Glycol in the Presence of Seeds and Poly(Vinyl Pyrrolidone). Chemistry of Materials.

[41] Xia Y, Xiong Y, Lim B, Skrabalak SE (2009). Shape-controlled synthesis of metal nanocrystals: simple chemistry meets complex physics?. Angew Chem Int Ed Engl.

[42] Bögels G, Meekes H, Bennema P, Bollen D (1999). Growth Mechanism of Vapor-Grown Silver Crystals:  Relation between Twin Formation and Morphology. The Journal of Physical Chemistry B.

[43] Sun YG, Gates B, Mayers B, Xia YN (2002). Crystalline silver nanowires by soft solution processing. Nano Lett..

[44] Chen C (2014). High-performance epoxy/silica coated silver nanowire composites as underfill material for electronic packaging. Composites Science and Technology.

[45] Yin Y, Lu Y, Sun Y, Xia Y (2002). Silver Nanowires Can Be Directly Coated with Amorphous Silica To Generate Well-Controlled Coaxial Nanocables of Silver/Silica. Nano Lett..

[46] Guo K (2013). Enhancement of properties of dye-sensitized solar cells by surface plasmon resonance of Ag nanowire core–shell structure in TiO2 films. Journal of Materials Chemistry A.

[47] Meenakshi P, Karthick R, Selvaraj M, Ramu S (2014). Investigations on reduced graphene oxide film embedded with silver nanowire as a transparent conducting electrode. Solar Energy Materials and Solar Cells.

[48] Kerker M (1985). The optics of colloidal silver: something old and something new. Journal of Colloid and Interface Science.

[49] Ditlbacher H (2005). Silver nanowires as surface plasmon resonators. Phys Rev Lett.

[50] Shegai T (2011). Unidirectional broadband light emission from supported plasmonic nanowires. Nano Lett.

[51] Geisler P, Krauss E, Razinskas G, Hecht B (2017). Transmission of Plasmons through a Nanowire. ACS Photonics.

[52] Saleh, B. E. A. & Teich, M. C. *Fundamentals of Photonics*. (Wiley, 2007).

[53] Chang DE, Sørensen AS, Demler EA, Lukin MD (2007). A single-photon transistor using nanoscale surface plasmons. Nature Physics.

[54] Kewes G (2016). A realistic fabrication and design concept for quantum gates based on single emitters integrated in plasmonic-dielectric waveguide structures. Scientific Reports.

[55] Ma R-M, Ota S, Li Y, Yang S, Zhang X (2014). Explosives detection in a lasing plasmon nanocavity. Nature Nanotechnology.

[56] Kewes, G., Binkowski, F., Burger, S., Zschiedrich, L. & Benson, O. Heuristic Modeling of Strong Coupling in Plasmonic Resonators. *ACS Photonics* (2018).

[57] Sun Y, Mayers B, Herricks T, Xia Y (2003). Polyol Synthesis of Uniform Silver Nanowires:  A Plausible Growth Mechanism and the Supporting Evidence. Nano Letters.

