# Supplementary material for: Silver nanowires with optimized silica coating as versatile plasmonic resonators
Source: Sci Rep. 2019 Mar 7;9:3859. doi: 10.1038/s41598-019-40380-5 (PMC6405757; doi:10.1038/s41598-019-40380-5)
Supplement: Supplementary file 1 — Supporting Information Silver nanowires with optimized silica coating as versatile plasmonic resonators [file 41598_2019_40380_MOESM1_ESM.pdf]

Supporting Information

Silver nanowires with optimized silica coating as versatile plasmonic resonators

Martin Rothe,[1] Yuhang Zhao,[2] Günter Kewes,[1] Zdravko Kochovski,[2] Wilfried Sigle,[3] Peter A. van Aken,[3] Christoph Koch,[4] Matthias Ballauff,[2,5] Yan Lu,[2,6] and Oliver Benson[1]

[1] Humboldt Universität zu Berlin & IRIS Adlershof, Nanooptics, Newtonstraße 15, 12489 Berlin, Germany
[2] Helmholtz Zentrum Berlin für Materialien und Energie, Institute of Soft Matter and Functional Materials, Hahn-Meitner-Platz 1, 14109 Berlin, Germany
[3] Stuttgart Center for Electron Microscopy, Max Planck Institute for Solid State Research, Heisenbergstr. 1, 70569 Stuttgart, Germany
[4] Humboldt Universität zu Berlin & IRIS Adlershof, Structure Research and Electron Microscopy, Newtonstraße 15, 12489 Berlin, Germany
[5] Humboldt Universität zu Berlin, Department of Physics, 12489 Berlin, Germany
[6] Institute of Chemistry, University of Potsdam, 14467 Potsdam, Germany

The purpose of this supplement is to present the results of the optical measurements for each single nanowire.

Fig. S1 to S2 present the bright-field and the dark-field images of each single pure silver nanowire investigated. The scattering from the end facets of the nanowires is clearly visible in the dark-field images. For further analysis, the scattered light from the front facet of the nanowires, i.e. the upper right end in the images, was studied spectroscopically. The measured spectra were background subtracted and normalized with the spectrum of the white light source. The resulting spectra are presented in the left column of fig. S3-S6. The white area around 650 nm was further investigated by transforming this part to frequency space and subsequent Fourier transformation. The result is presented in the center column. The right column presents a zoom from -200 fs to +200fs where peaks are expected for every multiple of the plasmon round trip time. The first peak, labeled by a black spot indicates the time for one plasmon round trip. At twice this time a second peak is expected. This position or a peak, if visible, is marked with a red spot. If a second peak is apparent the plasmon losses have been calculated. If not, the noise level was interpreted as upper limit for the second peak indicating a lower limit for the losses.

In analogy, Fig. S7 to S17 present the same results for silver nanowires coated with 8nm silica shell. The amount of samples has been increased to cover the greater distribution of lengths.

Fig. S18 to S20 show the ten studied silver nanowires with 24nm silica coating. Longitudinal modes are still visible but further effects reduce the quality of the evaluation method.

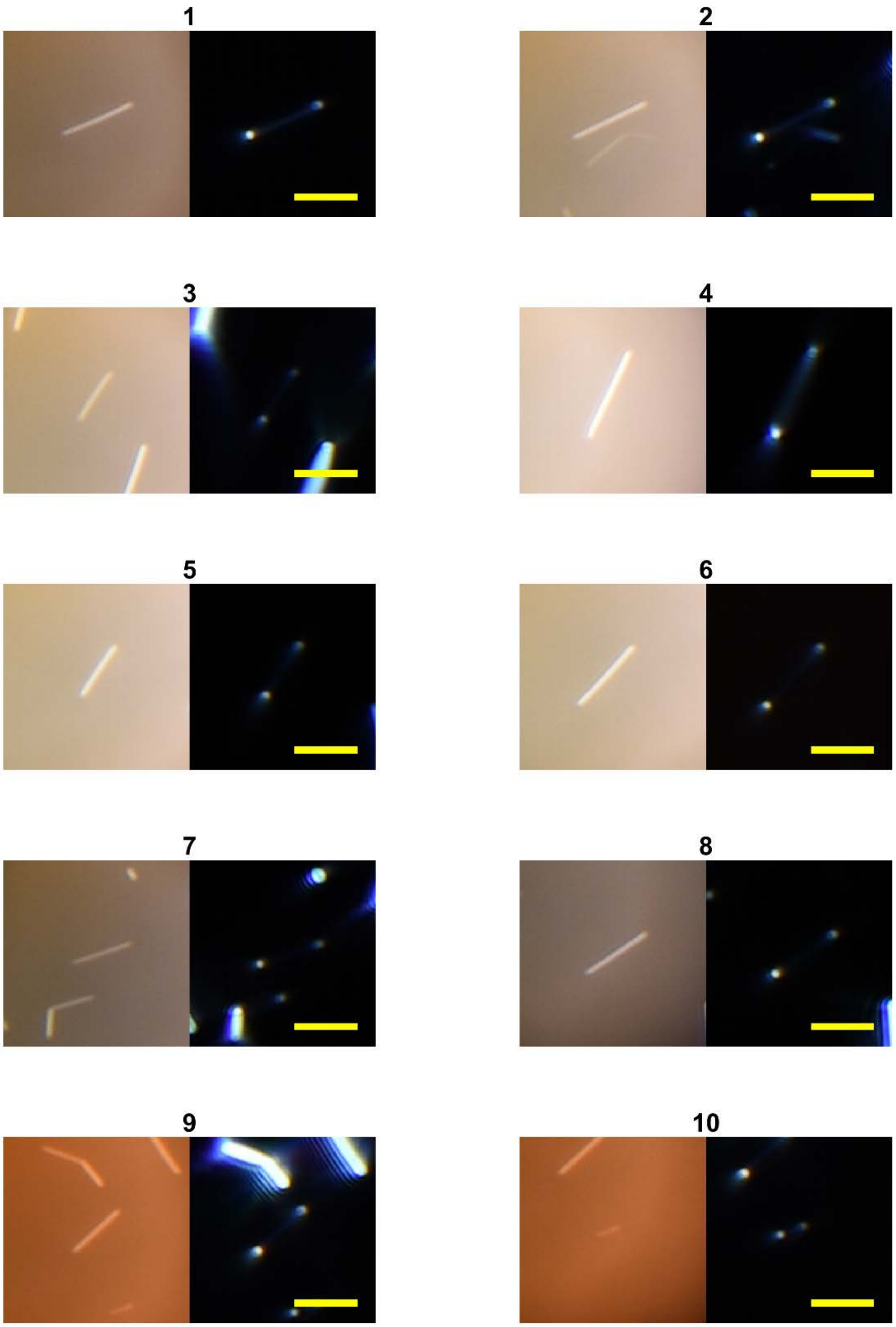

Figure S1. Pure silver nanowire samples 1 to 10 on coverglass are detected by bright-field imaging (left) and dark-field imaging (right), respectively. The scale bar is 5 μm.

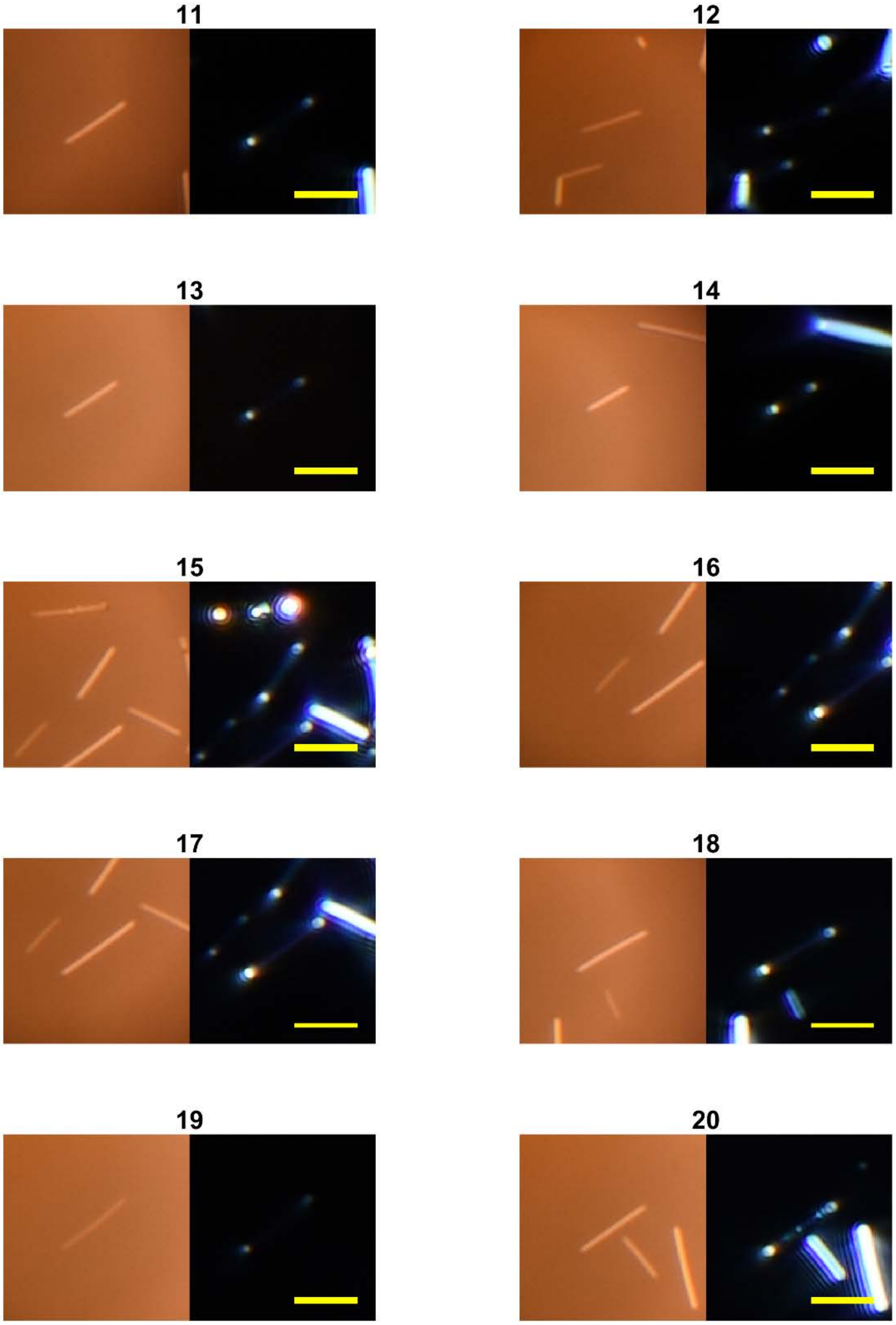

Figure S2. Pure silver nanowire samples 11 to 20 on coverglass are detected by bright-field imaging (left) and dark-field imaging (right), respectively. The scale bar is 5 μm.

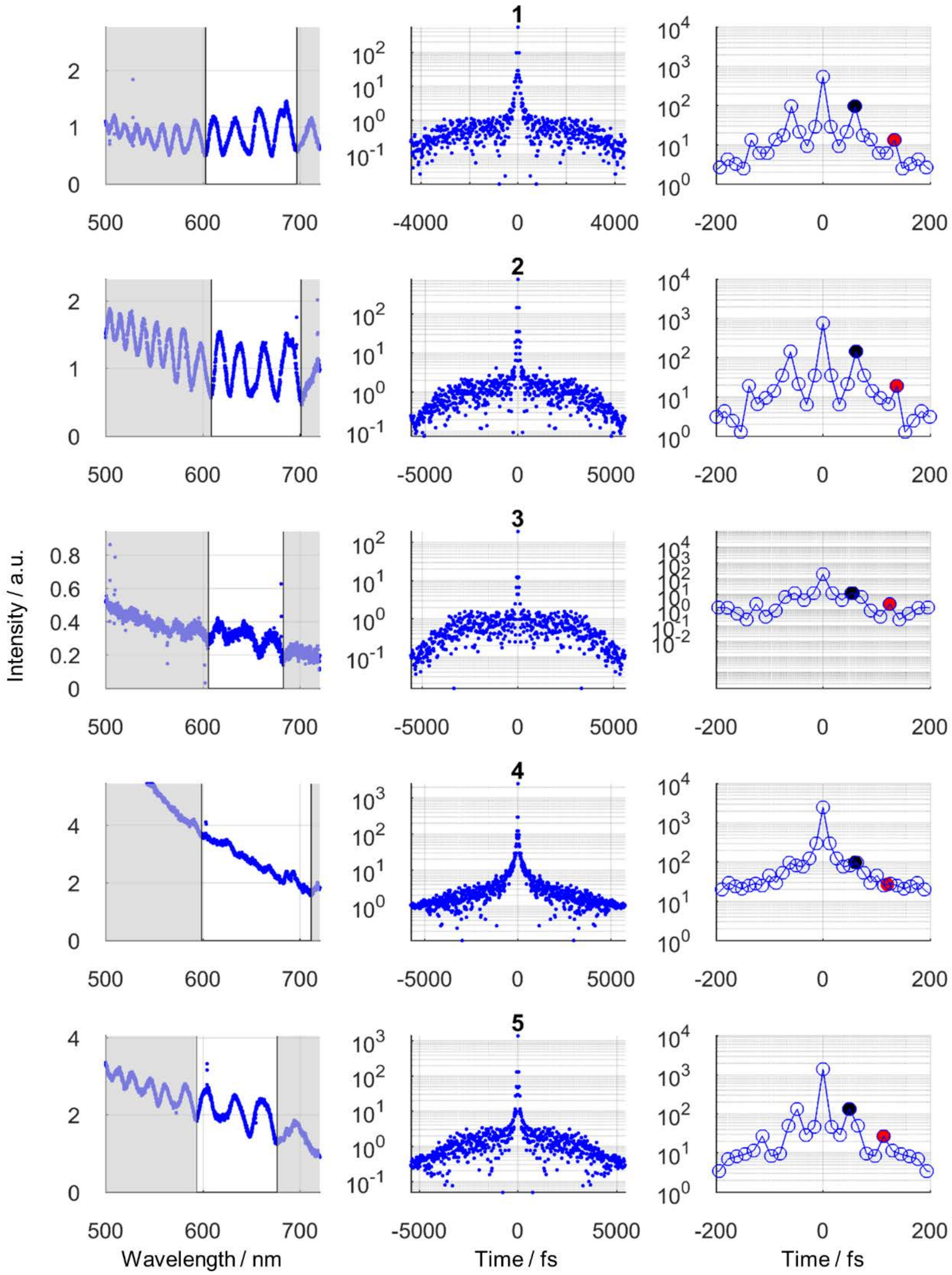

Figure S3. The normalized scattering spectrum (left) of the front facet of pure silver nanowire samples 1 to 5 has been cropped around 650 nm (left, white area) and Fourier transformed (middle) indicating the first (right, black dot) and the second (right, red dot) plasmon round trip.

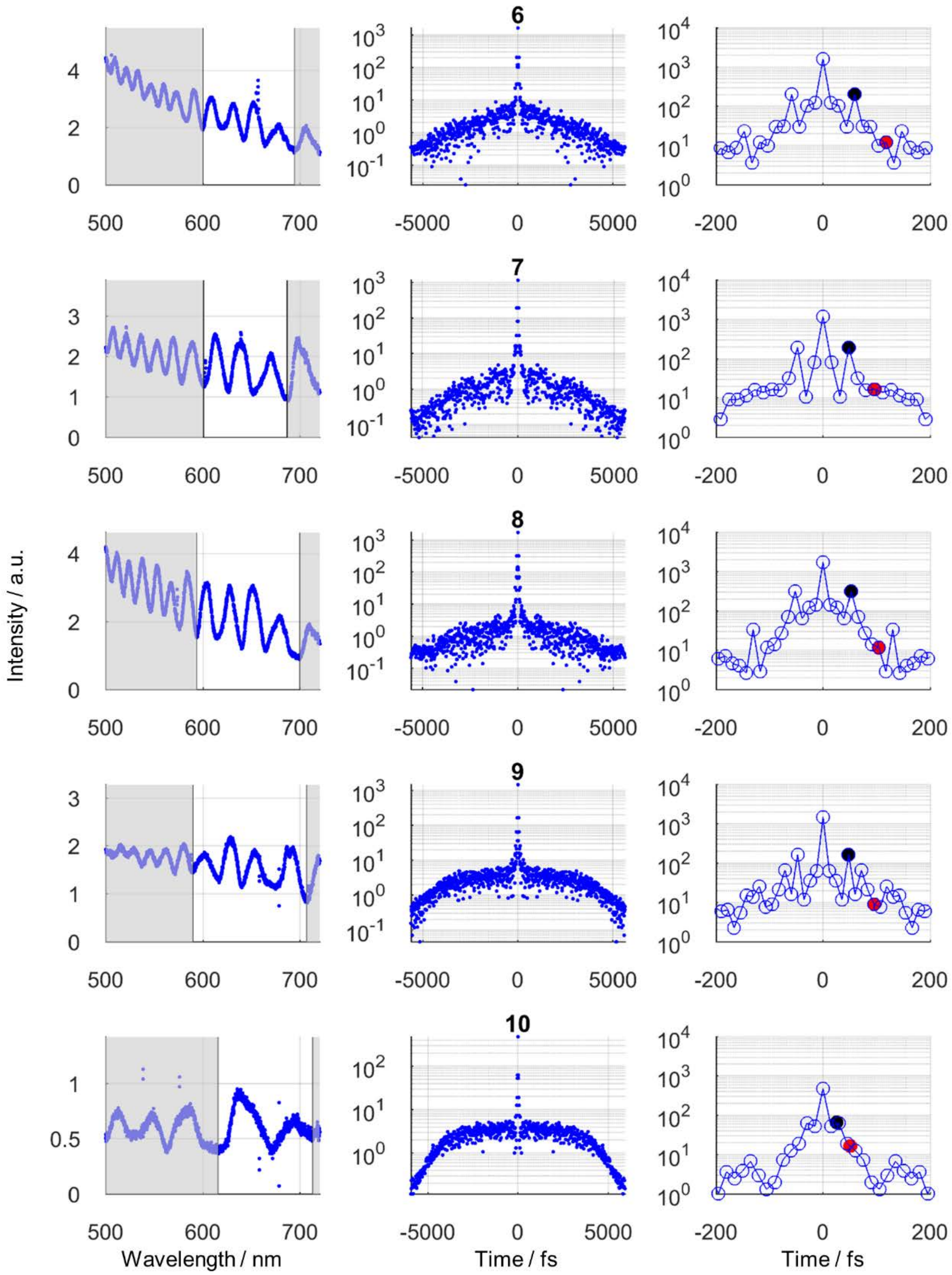

Figure S4. The normalized scattering spectrum (left) of the front facet of pure silver nanowire samples 6 to 10 has been cropped around 650 nm (left, white area) and Fourier transformed (middle) indicating the first (right, black dot) and the second (right, red dot) plasmon round trip.

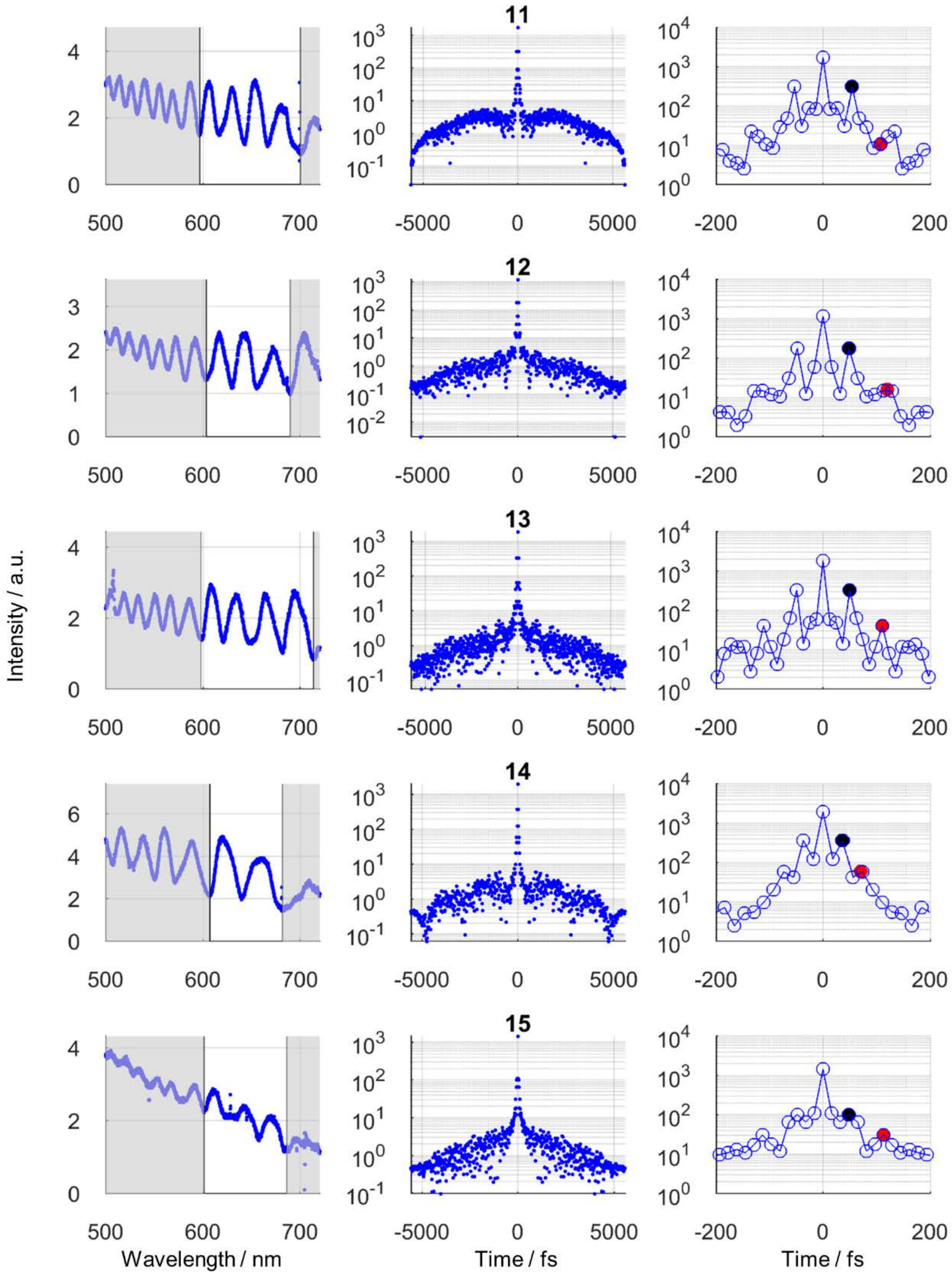

Figure S5. The normalized scattering spectrum (left) of the front facet of pure silver nanowire samples 11 to 15 has been cropped around 650 nm (left, white area) and Fourier transformed (middle) indicating the first (right, black dot) and the second (right, red dot) plasmon round trip.

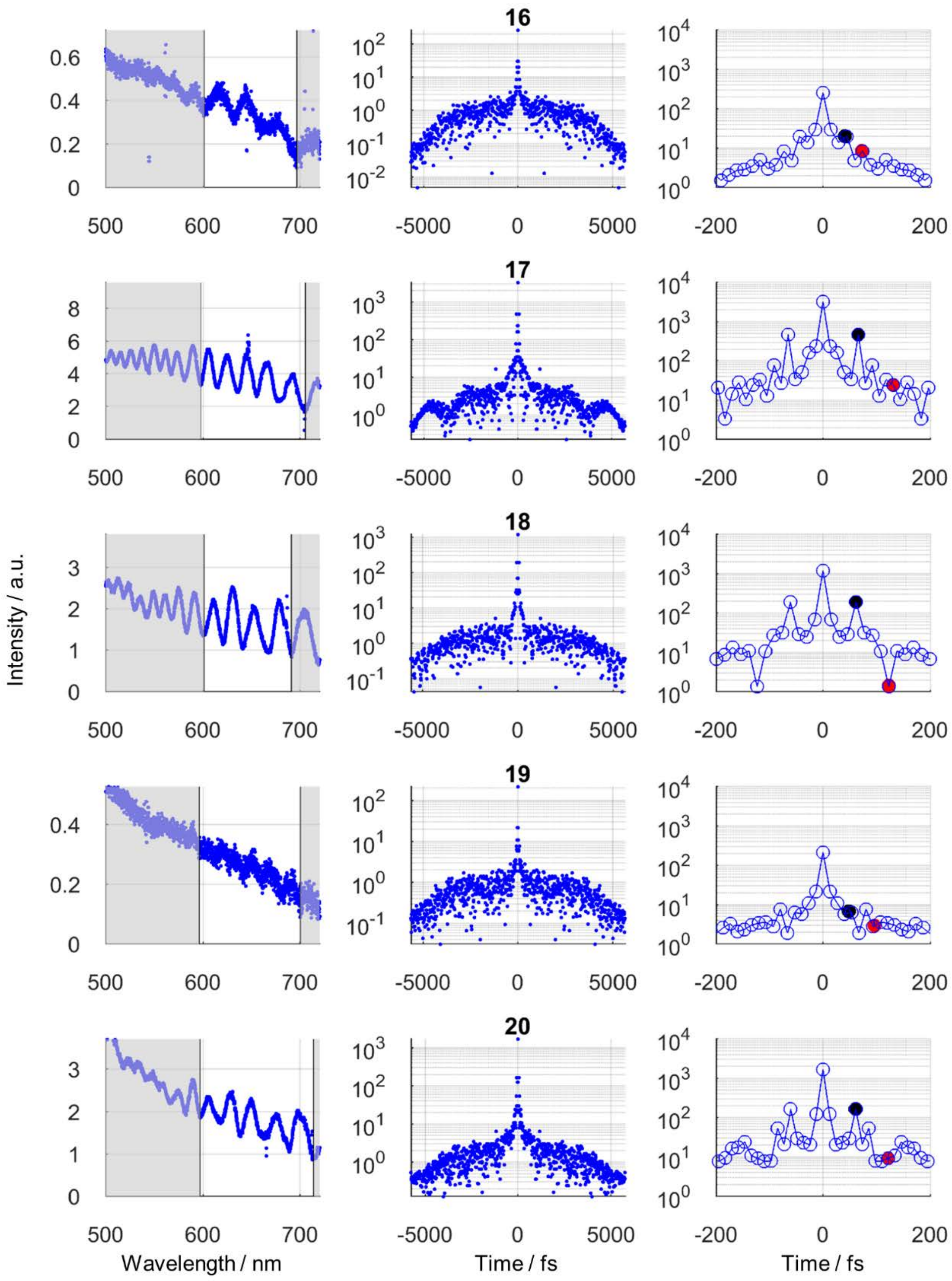

Figure S6. The normalized scattering spectrum (left) of the front facet of pure silver nanowire samples 16 to 20 has been cropped around 650 nm (left, white area) and Fourier transformed (middle) indicating the first (right, black dot) and the second (right, red dot) plasmon round trip.

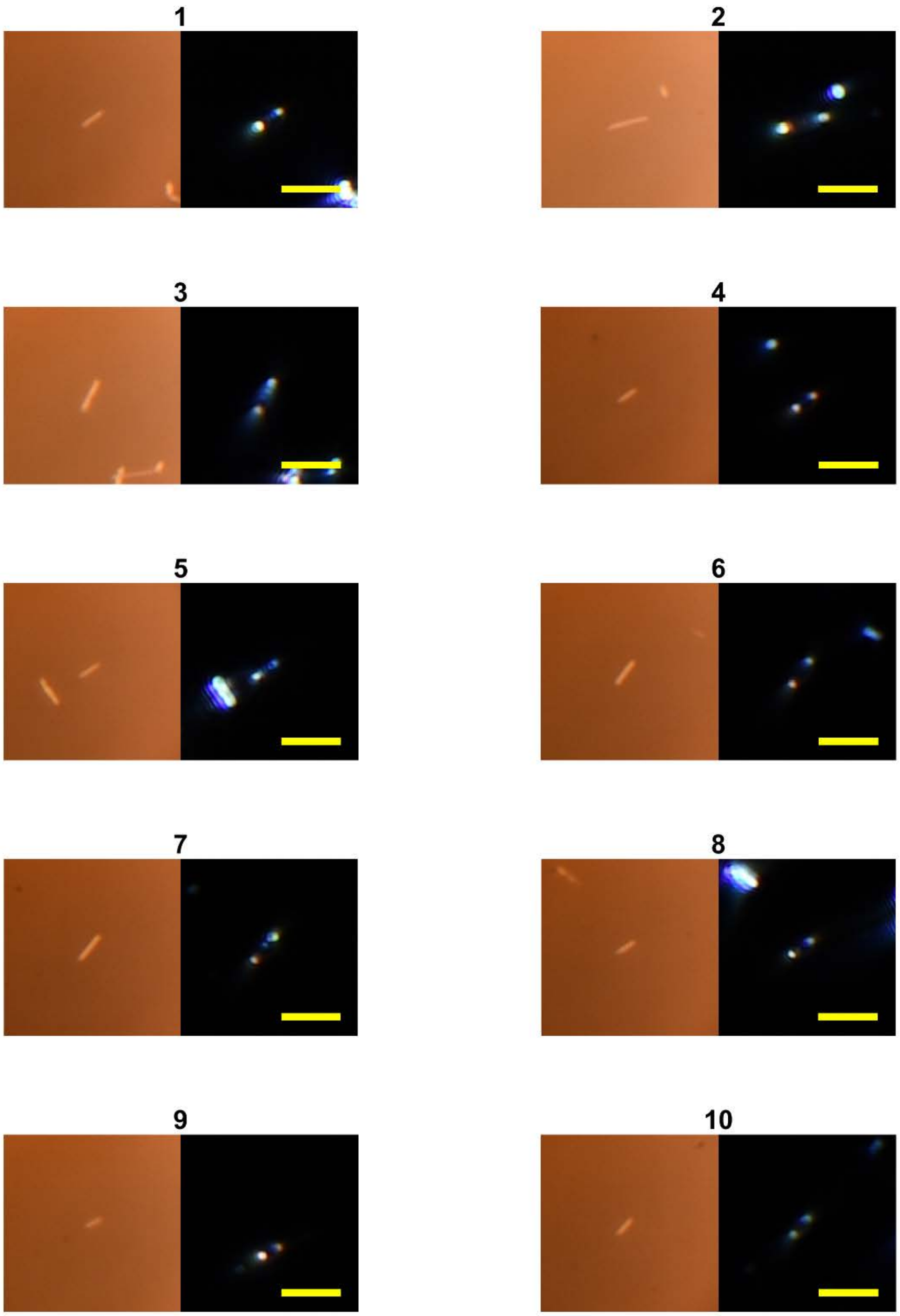

Figure S7. In analogy, silver nanowire with 8 nm silica coating samples 1 to 10 in bright-field imaging (left) and dark-field imaging (right), respectively. The scale bar is 5 μm.

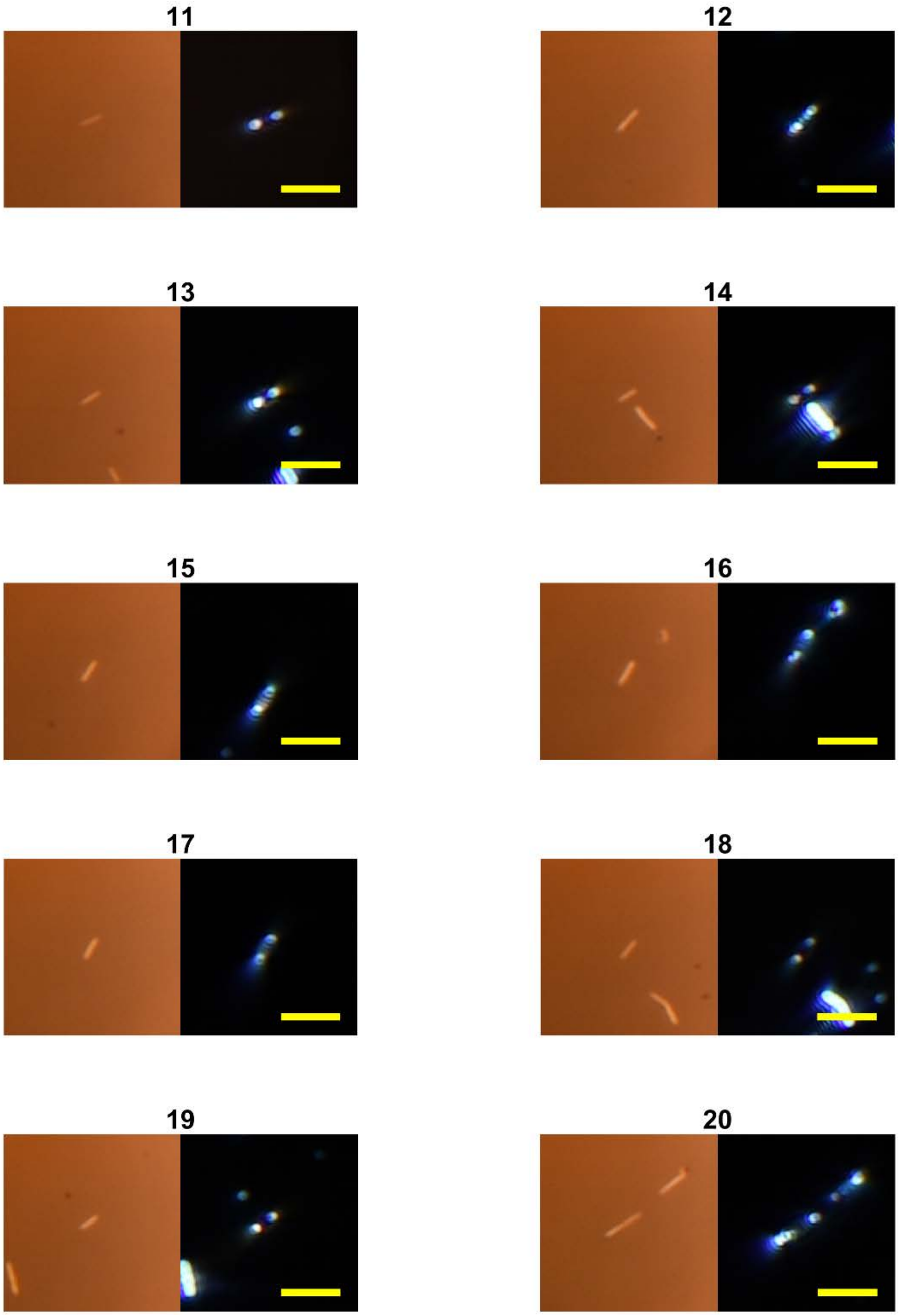

Figure S8. In analogy, silver nanowire with 8 nm silica coating samples 11 to 20 in bright-field imaging (left) and dark-field imaging (right), respectively. The scale bar is 5 μm.

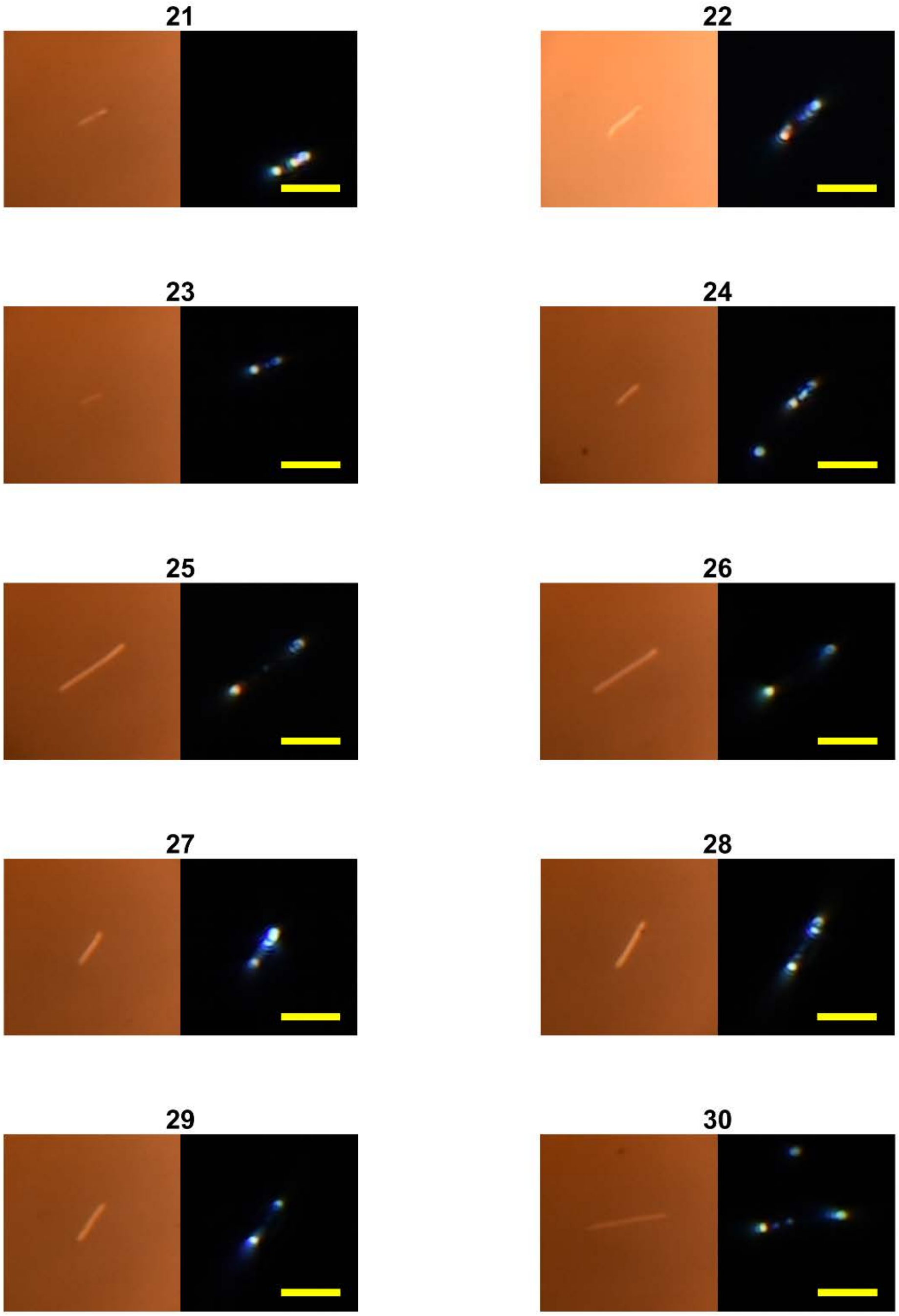

Figure S9. In analogy, silver nanowire with 8 nm silica coating samples 21 to 30 in bright-field imaging (left) and dark-field imaging (right), respectively. The scale bar is 5 μm.

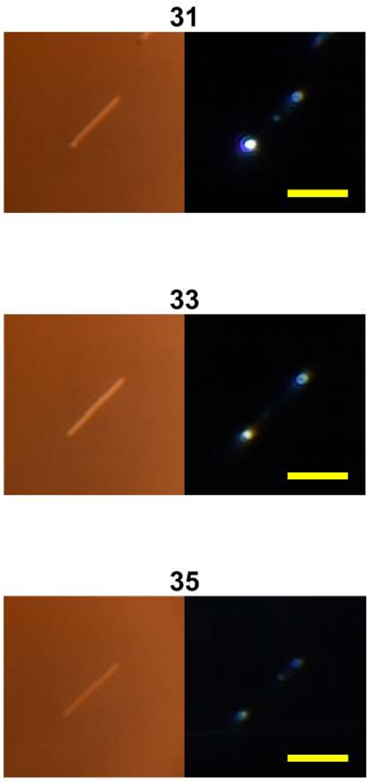

Figure S10. In analogy, silver nanowire with 8 nm silica coating samples 31 to 35 in bright-field imaging (left) and dark-field imaging (right), respectively. The scale bar is 5 μm.

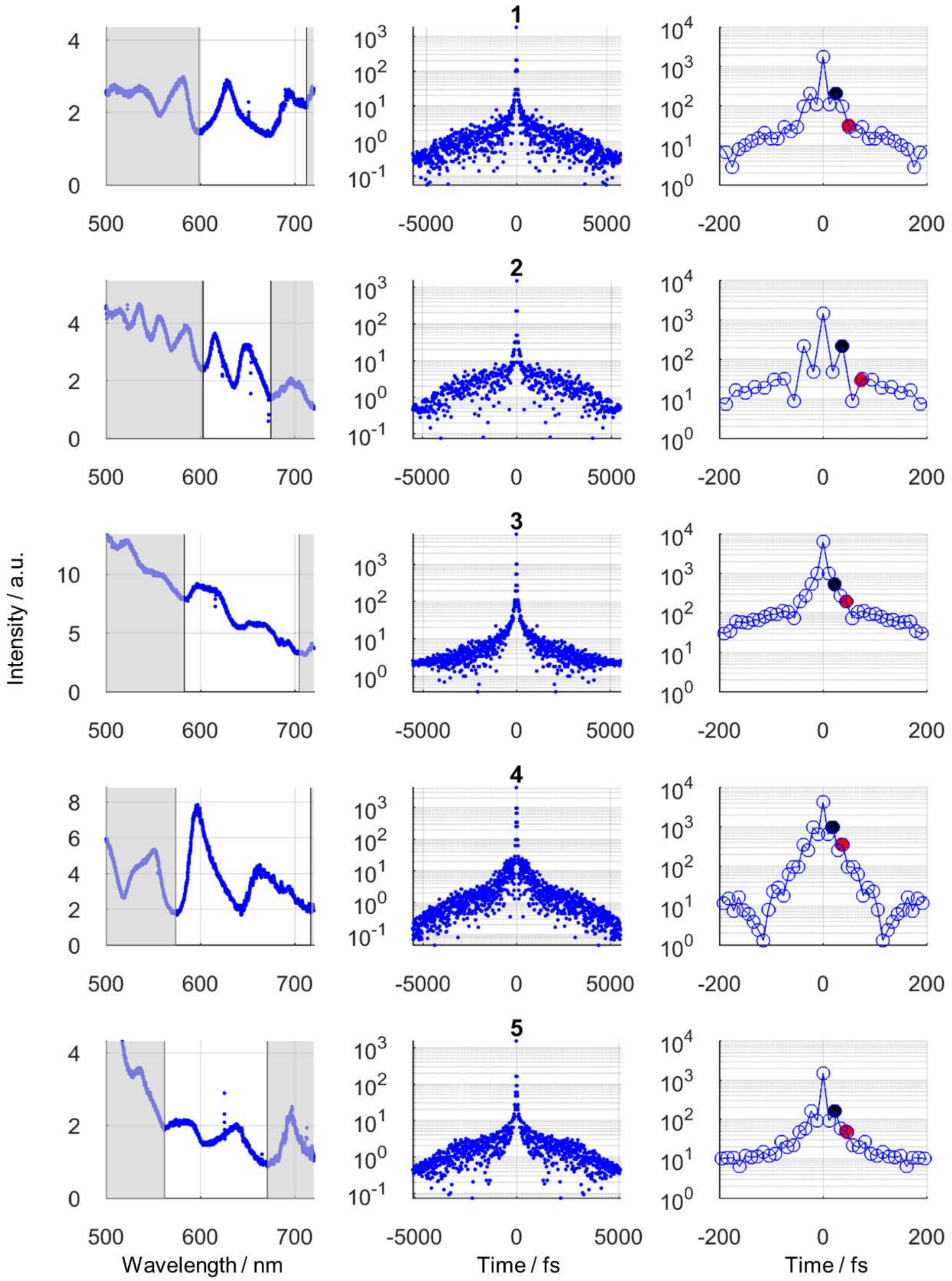

Figure S11. In analogy, the normalized scattering spectrum (left) of silver nanowire with 8 nm silica coating samples 1 to 5, Fourier transformation (middle) and indicated first (right, black dot) and second (right, red dot) plasmon round trip.

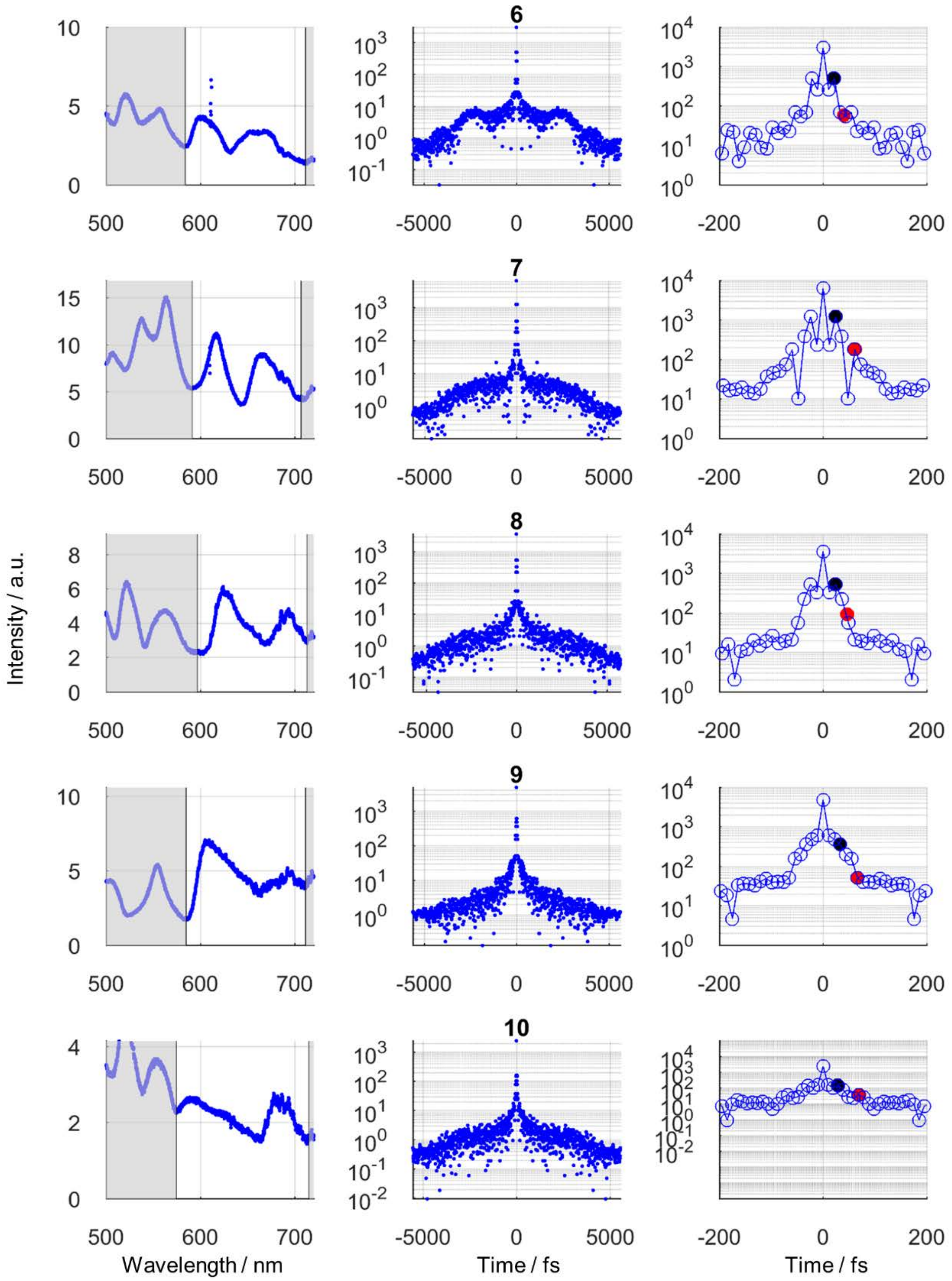

Figure S12. In analogy, the normalized scattering spectrum (left) of silver nanowire with 8 nm silica coating samples 6 to 10, Fourier transformation (middle) and indicated first (right, black dot) and second (right, red dot) plasmon round trip.

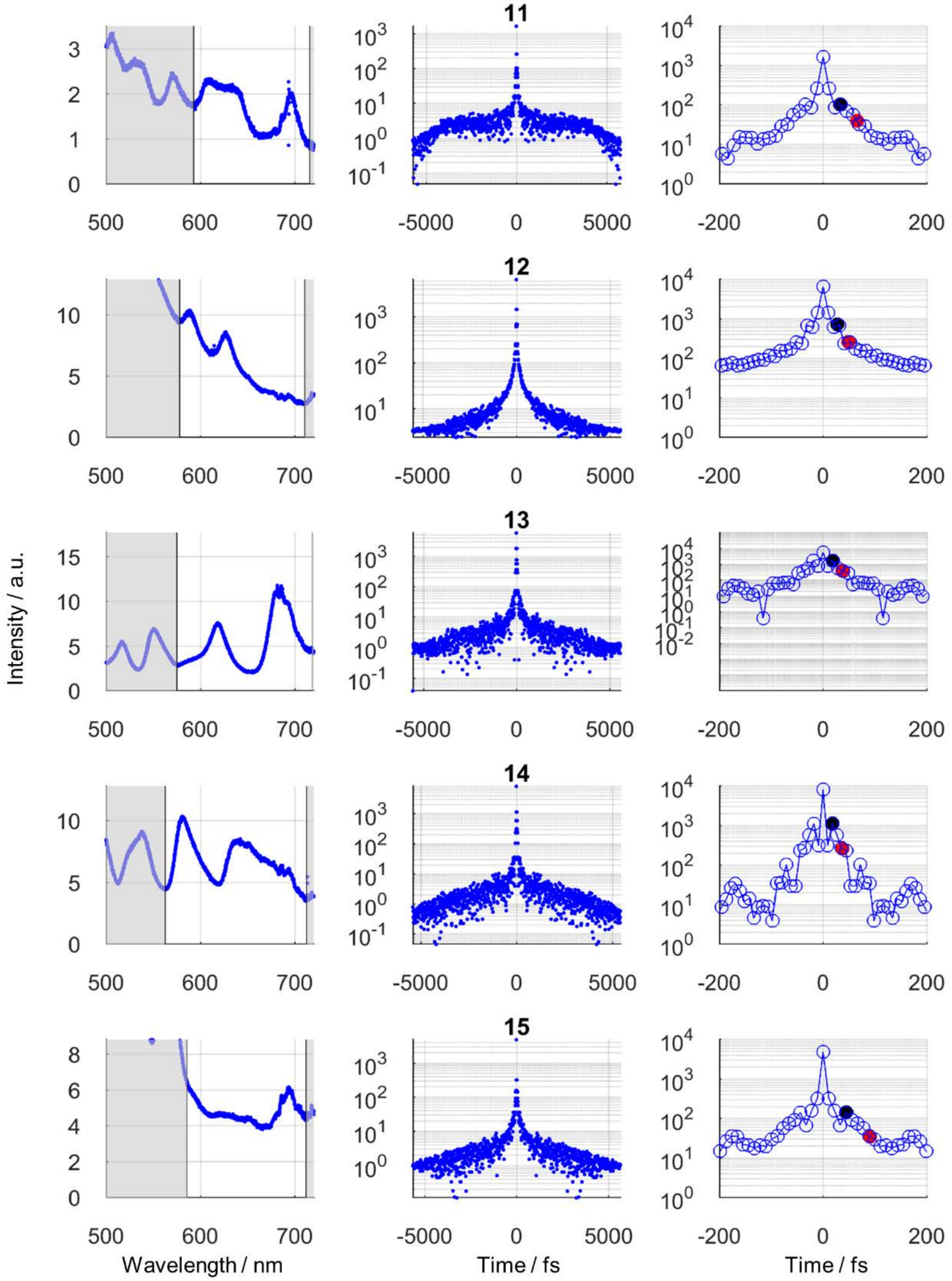

Figure S13. In analogy, the normalized scattering spectrum (left) of silver nanowire with 8 nm silica coating samples 11 to 15, Fourier transformation (middle) and indicated first (right, black dot) and second (right, red dot) plasmon round trip.

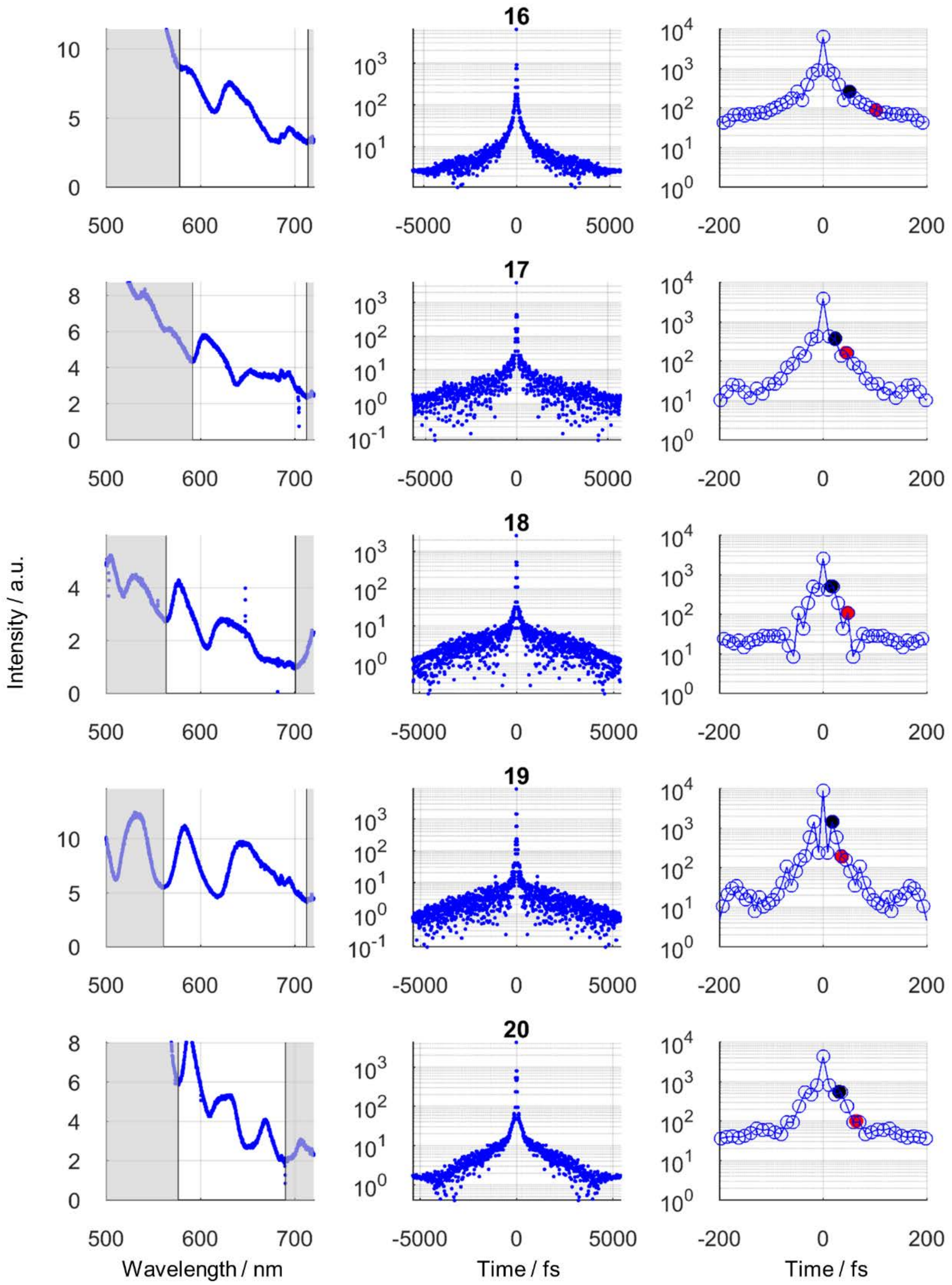

Figure S14. In analogy, the normalized scattering spectrum (left) of silver nanowire with 8 nm silica coating samples 16 to 20, Fourier transformation (middle) and indicated first (right, black dot) and second (right, red dot) plasmon round trip.

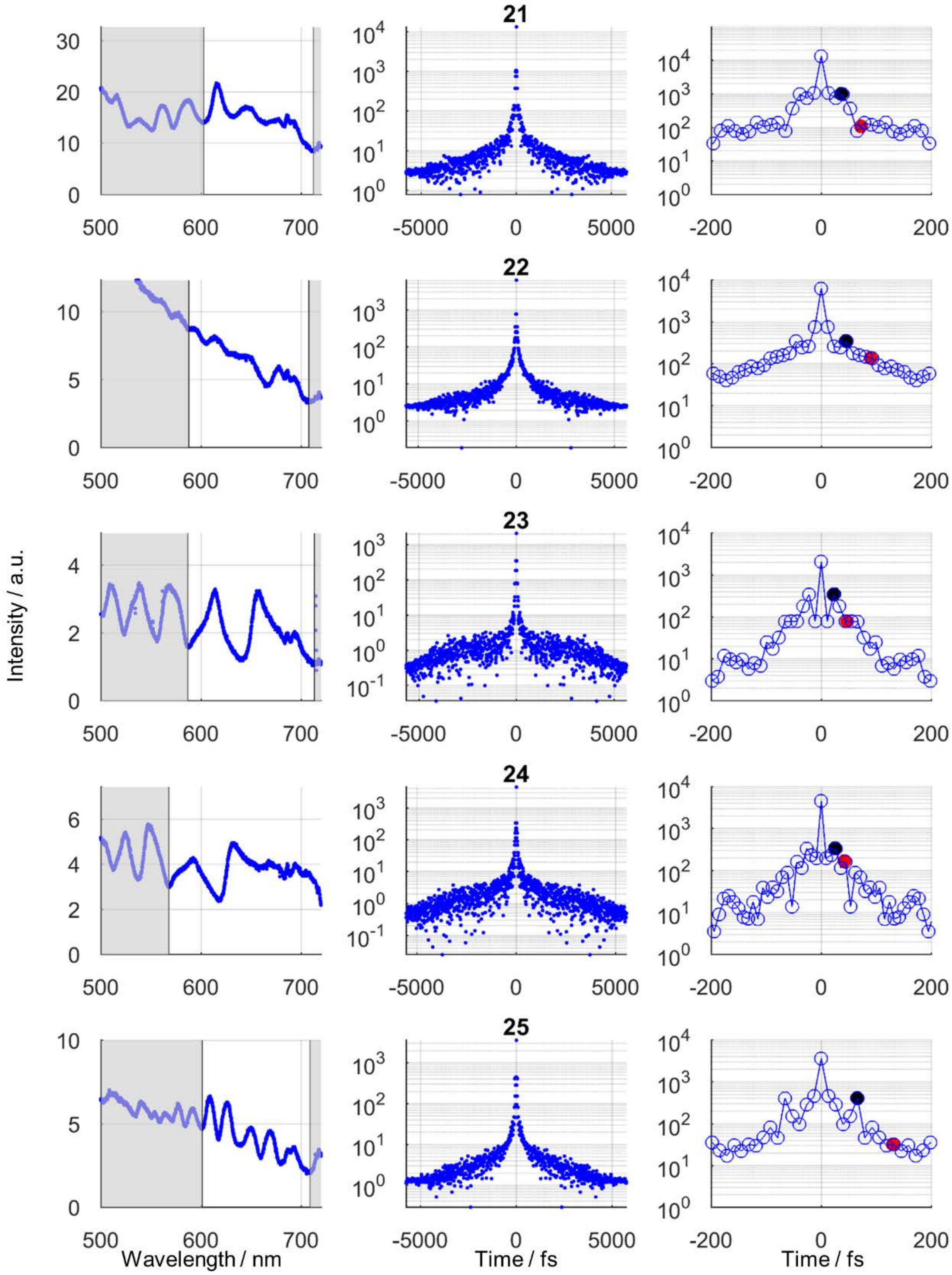

Figure S15. In analogy, the normalized scattering spectrum (left) of silver nanowire with 8 nm silica coating samples 21 to 25, Fourier transformation (middle) and indicated first (right, black dot) and second (right, red dot) plasmon round trip.

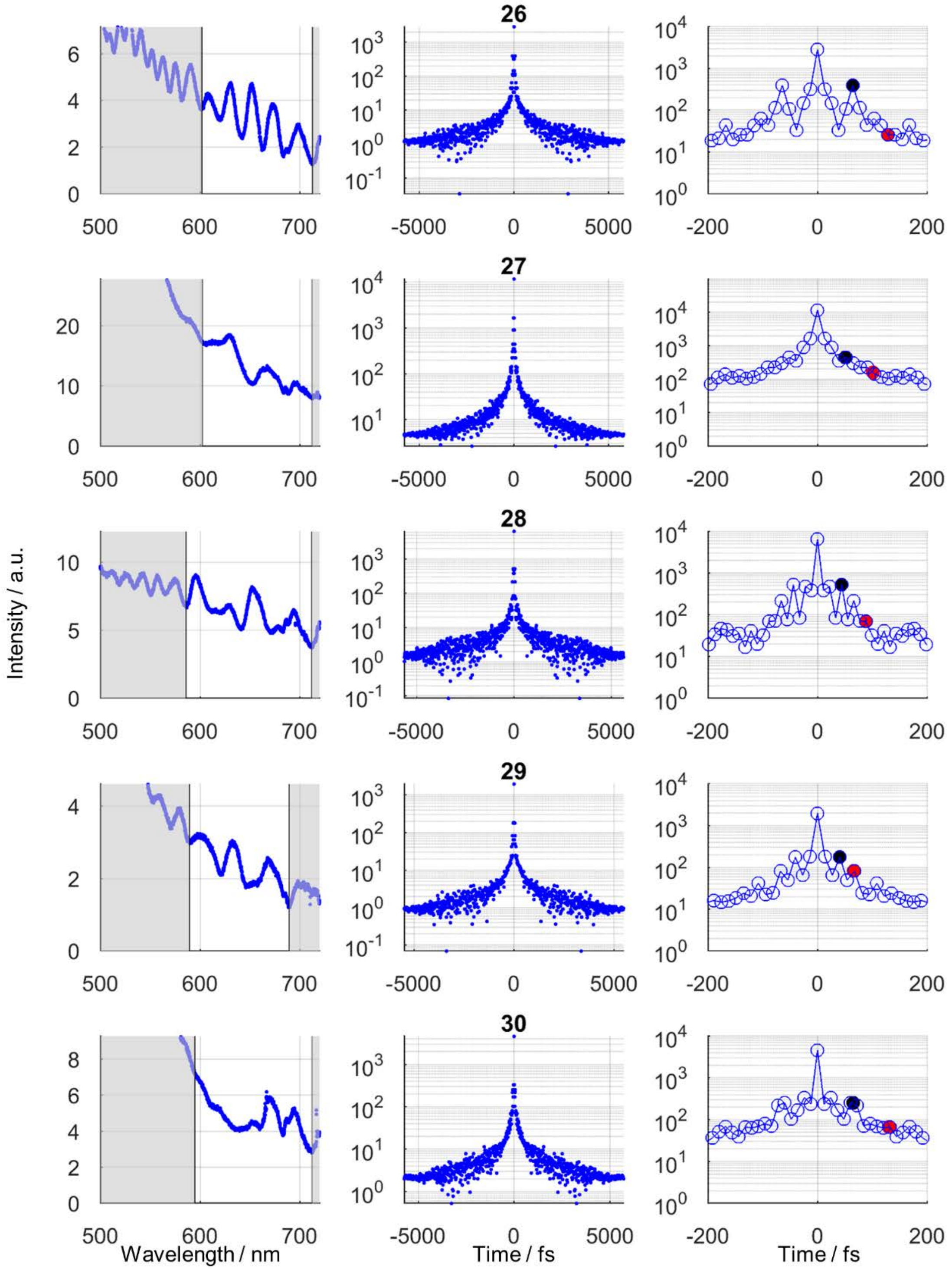

Figure S16. In analogy, the normalized scattering spectrum (left) of silver nanowire with 8 nm silica coating samples 26 to 30, Fourier transformation (middle) and indicated first (right, black dot) and second (right, red dot) plasmon round trip.

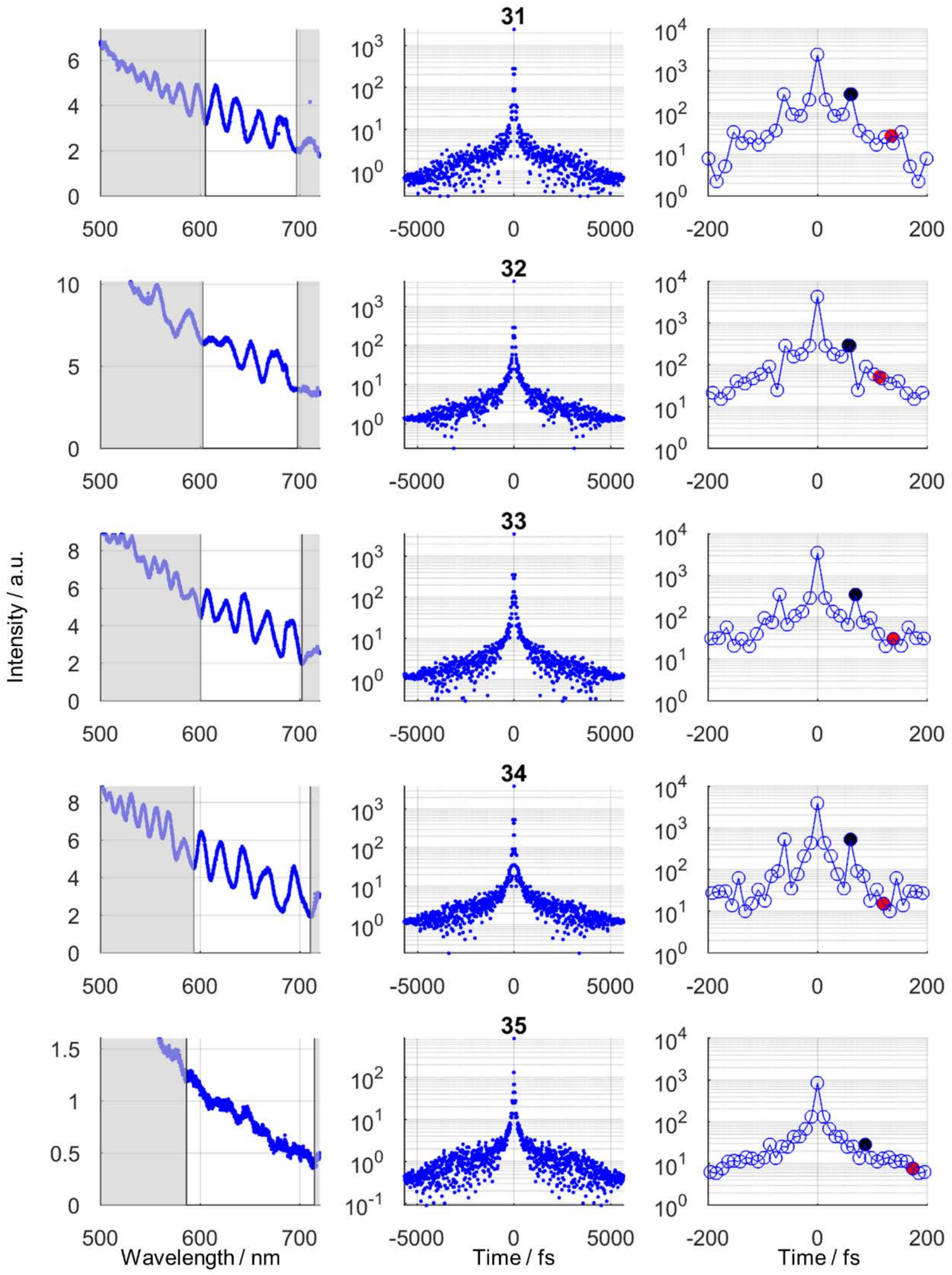

Figure S17. In analogy, the normalized scattering spectrum (left) of silver nanowire with 8 nm silica coating samples 30 to 35, Fourier transformation (middle) and indicated first (right, black dot) and second (right, red dot) plasmon round trip.

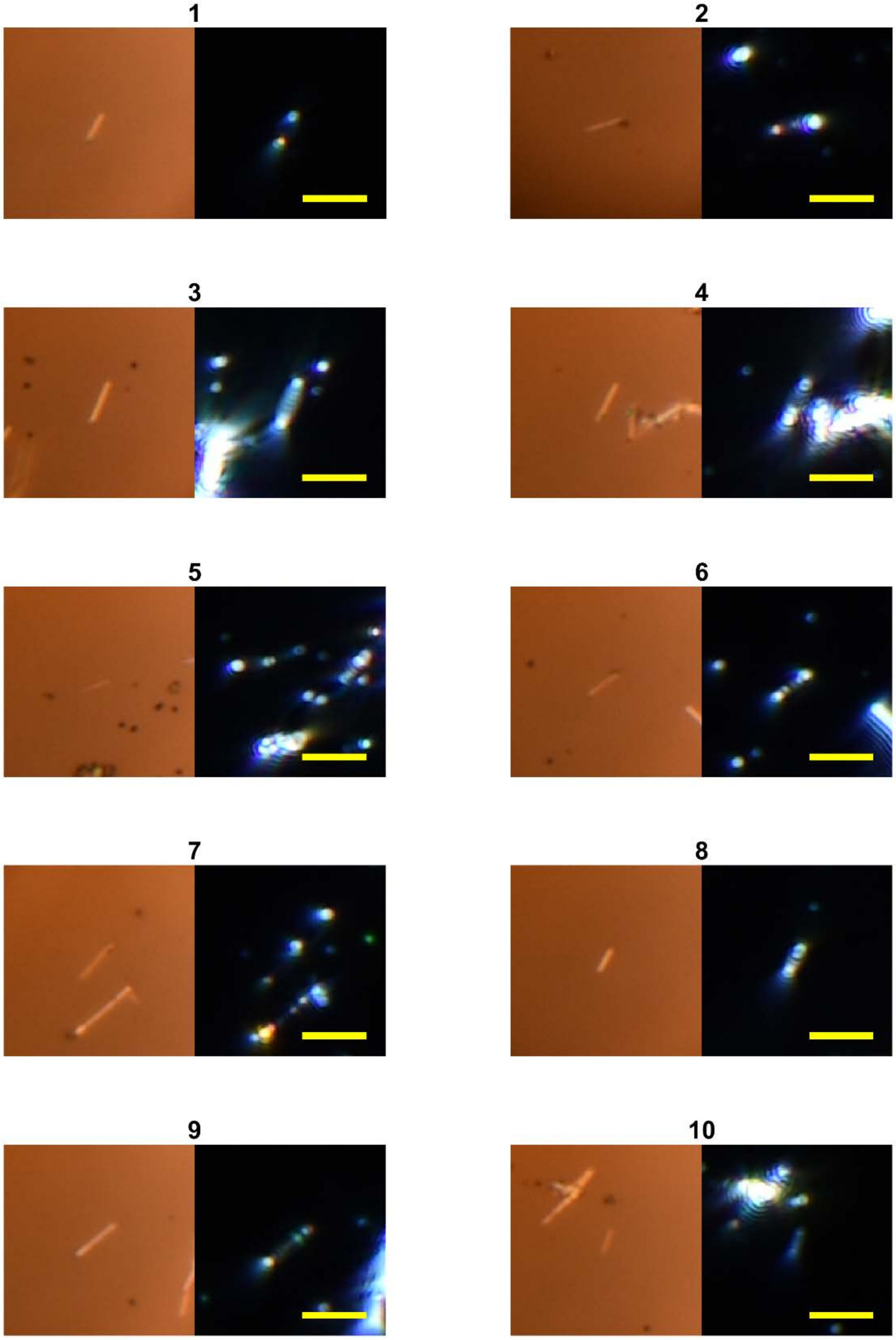

Figure S18. To present limitations in the preparation and the optical method, silver nanowire with 24 nm silica coating samples were studied in bright-field imaging (left) and dark-field imaging (right), respectively. The scale bar is 5 μm.

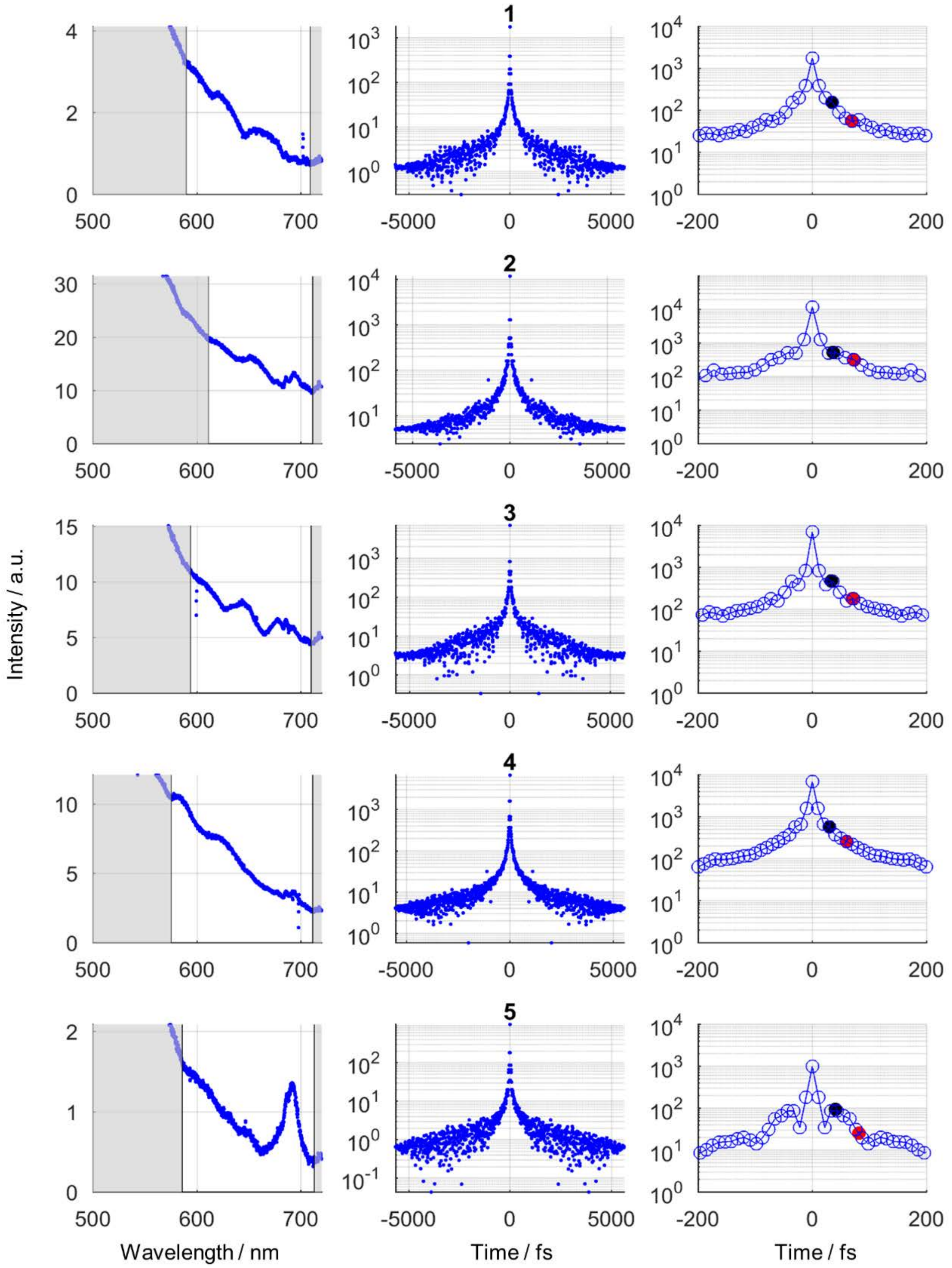

Figure S19. In analogy, the normalized scattering spectrum (left) of silver nanowire with 24 nm silica coating samples 1 to 5, Fourier transformation (middle) and indicated first (right, black dot) and second (right, red dot) plasmon round trip.

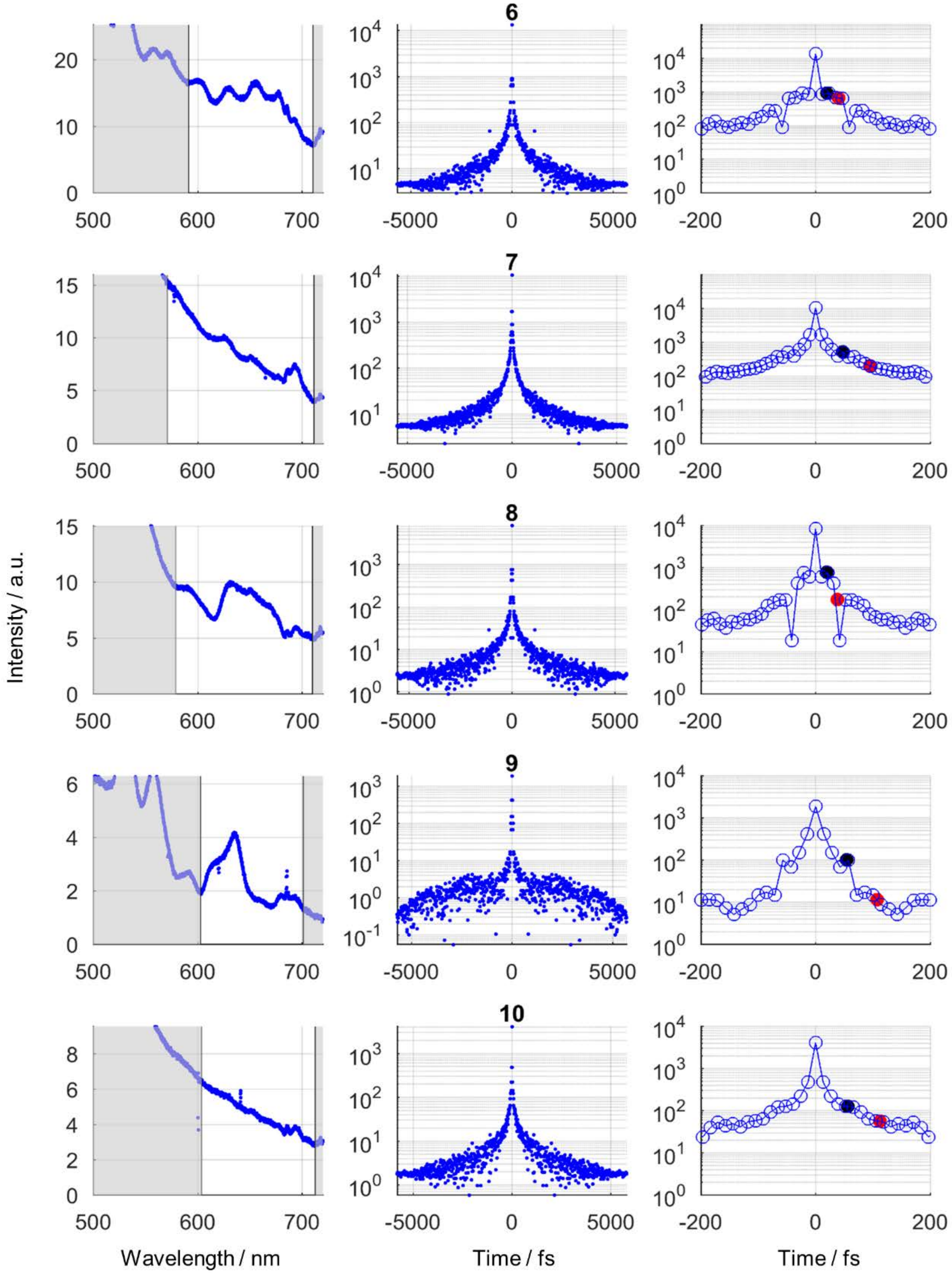

Figure S20. In analogy, the normalized scattering spectrum (left) of silver nanowire with 24 nm silica coating samples 5 to 10, Fourier transformation (middle) and indicated first (right, black dot) and second (right, red dot) plasmon round trip.
